# Information-theoretic analyses of neural data to minimize the effect of researchers’ assumptions in predictive coding studies

**DOI:** 10.1371/journal.pcbi.1011567

**Published:** 2023-11-17

**Authors:** Patricia Wollstadt, Daniel L. Rathbun, W. Martin Usrey, André Moraes Bastos, Michael Lindner, Viola Priesemann, Michael Wibral

**Affiliations:** 1 MEG Unit, Brain Imaging Center, Goethe University, Frankfurt/Main, Germany; 2 Center for Neuroscience, University of California, Davis, California, United States of America; 3 Center for Ophthalmology, University of Tübingen, Tübingen, Germany; 4 Department of Neurobiology, Physiology, and Behavior, University of California, Davis, California, United States of America; 5 Department of Psychology and Vanderbilt Brain Institute, Vanderbilt University, Nashville, Tennessee, United States of America; 6 Campus Institute for Dynamics of Biological Networks, University of Göttingen, Göttingen, Germany; 7 Max Planck Institute for Dynamics and Self-Organization, Göttingen, Germany; RU Nijmegen Donders Institute: Radboud Universiteit Donders Institute for Brain Cognition and Behaviour, NETHERLANDS

## Abstract

Studies investigating neural information processing often implicitly ask both, which processing strategy out of several alternatives is used and how this strategy is implemented in neural dynamics. A prime example are studies on predictive coding. These often ask whether confirmed predictions about inputs or prediction errors between internal predictions and inputs are passed on in a hierarchical neural system—while at the same time looking for the neural correlates of coding for errors and predictions. If we do not know exactly what a neural system predicts at any given moment, this results in a circular analysis—as has been criticized correctly. To circumvent such circular analysis, we propose to express information processing strategies (such as predictive coding) by local information-theoretic quantities, such that they can be estimated directly from neural data. We demonstrate our approach by investigating two opposing accounts of predictive coding-like processing strategies, where we quantify the building blocks of predictive coding, namely predictability of inputs and transfer of information, by local active information storage and local transfer entropy. We define testable hypotheses on the relationship of both quantities, allowing us to identify which of the assumed strategies was used. We demonstrate our approach on spiking data collected from the retinogeniculate synapse of the cat (*N* = 16). Applying our local information dynamics framework, we are able to show that the synapse codes for predictable rather than surprising input. To support our findings, we estimate quantities applied in the partial information decomposition framework, which allow to differentiate whether the transferred information is primarily bottom-up sensory input or information transferred conditionally on the current state of the synapse. Supporting our local information-theoretic results, we find that the synapse preferentially transfers bottom-up information.

## Author summary

Many neuroscience studies investigate how neural systems, e.g., neural circuits or cortical areas, process information. Popular theories propose that such systems operate by constantly predicting future sensory inputs from internal models built from previous inputs. Here, opposing accounts exist on how these predictions and actual inputs are reconciled to improve future predictions. One popular theory proposes that neural systems make use of prediction errors to update the internal model, while other theories propose that predominantly correctly predicted information is used. Testing which of these two strategies is actually employed by neural systems is conceptually difficult because it requires to define beforehand when a prediction error should occur and by what changes in the data such an occurrence should be indicated. This knowledge is typically not available, making it difficult to test both strategies against each other. Instead, we propose to use information-theoretic quantities to make central information processing concepts such as predictions and prediction errors measurable from data, which in turn allows to formulate testable hypotheses on information processing carried out by neural systems. We demonstrate our approach on data recorded at retinal synapses of the cat and successfully describe which of the two processing strategies is used.

## Introduction

Predictive coding as a theory arguably dominates today’s scientific discourse on how the cortex works [1–3]. Importantly, it is positioned as a theory of general cortical function—yet, empirical tests so far are limited to situations with an explicitly predictive experimental context, simply to allow for a meaningful analysis. In other words, to find and understand the neurophysiological correlates of predictions and errors, experiments posit a priori, when and what is being predicted in which brain region. There are three problems with this approach: first, knowing what is being predicted when and where in the brain seems to require already a fair understanding of how the brain, or the cortex, works—which may not generally be available yet. Second, trying to acquire some of the necessary knowledge post-hoc, runs the real risk of involuntarily producing a circular analysis or argument, or a “just-so story” (as it is called e.g. in section 4.1 of [4]). Third, restricting empirical tests of a general theory to experimental contexts that are explicitly designed with predictions in mind, in a strict sense, prohibits conclusions about the applicability of that theory in other contexts. One might provocatively frame this third problems as: “Is the cortex doing predictive coding when we don’t test it?” Last but not least, the latter restriction to dedicated experimental designs excludes testing (and possibly refuting) the theory by drawing on the vast majority of empirical neurophysiological data, i.e., all data that were obtained with a focus on descriptions of cortical function(s) other than predictive coding, which seems like a waste of available empirical evidence.

In this paper, we introduce an information-theoretic framework for testing predictive coding theories by translating the concepts of predictability, predictions, and prediction errors (surprise) into information-theoretic quantities measurable from data. Based on these information-theoretic formulations, we describe how to test by pure information-theoretical means whether a neural processing element (neuron, or a small circuit) takes part in a predictive coding-like computation. Our novel method is in principle applicable without knowledge of the intentions of the experimenter, given some weak constraints on the data themselves. Our method rests on the simple idea that a neural processing element that codes for prediction errors should exhibit high information transfer at moments when its input is surprising (i.e. fundamentally unpredictable), and vice versa [5]. On the other hand, a processing element coding for the predictable information in its inputs should exhibit high information transfer at times when this predictable information is high.

In the following we formalize this idea in the mathematical context of local information dynamics [6] and partial information decomposition [7].

## Methods

### Ethics statement

All surgical and experimental procedures were performed with the approval of the Animal Care and Use Committee at the University of California, Davis. For details see [8].

### Local information dynamics

As local information dynamics [6] is a relatively recent subfield of information theory, such that the inspection of local information quantities is not yet widely applied, we (re-)introduce these concepts with some detail. In this exposition we try to balance a concise and intuitive presentation of the material with mathematical rigor, necessarily sacrificing some of the latter. For a more detailed introduction see [5, 6].

### Local mutual information

For the purpose of this study it is best to understand the mutual information, *I*(*X*: *Y*), between two random variables by stating that if one variable *X* has information about another variable, *Y*, then *X* and *Y* can not be statistically independent. This point of view will help to understand why each individual term, $\text{log}\;\frac{p(x,y)}{p(x)p(y)}$, that contributes to the summation in the mutual information:

$$\begin{array}{c} I\left(X:Y\right)=\sum_{x,y\in A_{X,Y}}p\left(x,y\right)\;\text{log}\;\frac{p(x,y)}{p(x)p(y)}\;, \end{array}$$ (1)

can be soundly interpreted on its own. By $x\in A_{X}$ and $y\in A_{Y}$, we denote individual realizations of random variables *X* and *Y*, and we write *p*(*x*) as a shorthand for the probability *p*(*X* = *x*). We start our explanation with the definition of statistical independence of *X* and *Y* as:

$$\begin{array}{c} p\left(x,y\right)=p\left(x\right)p\left(y\right)\;\forall x,y\in A_{X,Y}\;, \end{array}$$ (2)

meaning that the equation *p*(*x*, *y*) = *p*(*x*)*p*(*y*) must hold for *all* pairs of realizations (*x*, *y*). If the above equation is violated for any pair (*x*, *y*) then this pair contributes to a deviation from independence. As per our initial statement on the relation of independence and information, this pair then also contributes to the information that *X* holds about *Y*, and vice versa.

To measure how much independence is violated locally by the pair (*x*, *y*), we can take the ratio of both sides of Eq 2. Now, independence, or the absence of mutual information, is equivalent to:

$$\begin{array}{c} \frac{p(x,y)}{p(x)p(y)}=1\;\forall x,y\in A_{X,Y}\;. \end{array}$$ (3)

A deviation of this ratio from 1 for any pair (*x*, *y*) indicates a deviation from independence, i.e., the presence of information in the realization of one variable about the realization of the other. Obviously one would like a measure of this information itself to be zero in the absence of information, i.e., at independence. This can be achieved by taking the logarithm of Eq 3:

$$\begin{array}{c} \text{log}\;\frac{p(x,y)}{p(x)p(y)}=0\;\forall x,y\in A_{X,Y}\;. \end{array}$$ (4)

Inspecting Eq 4 and comparing to the definition of the mutual information in Eq 1, we now see that the mutual information is nothing but the weighted average of the individual deviations from independence, measured on a logarithmic scale. More importantly the above derivation of the mutual information demonstrates that each individual term has a well defined and interpretable meaning. These individual terms define the local mutual information in a pair of realizations, *i*(*x*, *y*):

$$\begin{array}{c} i\left(x:y\right)=\text{log}\;\frac{p(x,y)}{p(x)p(y)}=\text{log}\;\frac{p(x|y)}{p(x)}\;. \end{array}$$ (5)

Analogously, the local conditional mutual information between two variables in the context of a third is given by:

$$\begin{array}{c} i\left(x:y|z\right)=\text{log}\;\frac{p(x,y|z)}{p(x|z)p(y|z)}=\text{log}\;\frac{p(x|y,z)}{p(x|z)}\;. \end{array}$$ (6)

We note that the local interpretation introduced here is closely related to the way Fano originally derived the mutual information [9]. In addition, we note that the local mutual information and the local conditional mutual information can be negative—in contrast to the (average) mutual information which is always positive or zero. We will explain this fact in detail, and make use of it, further below.

### Local active information storage as locally predictable information

**Fig 1:**
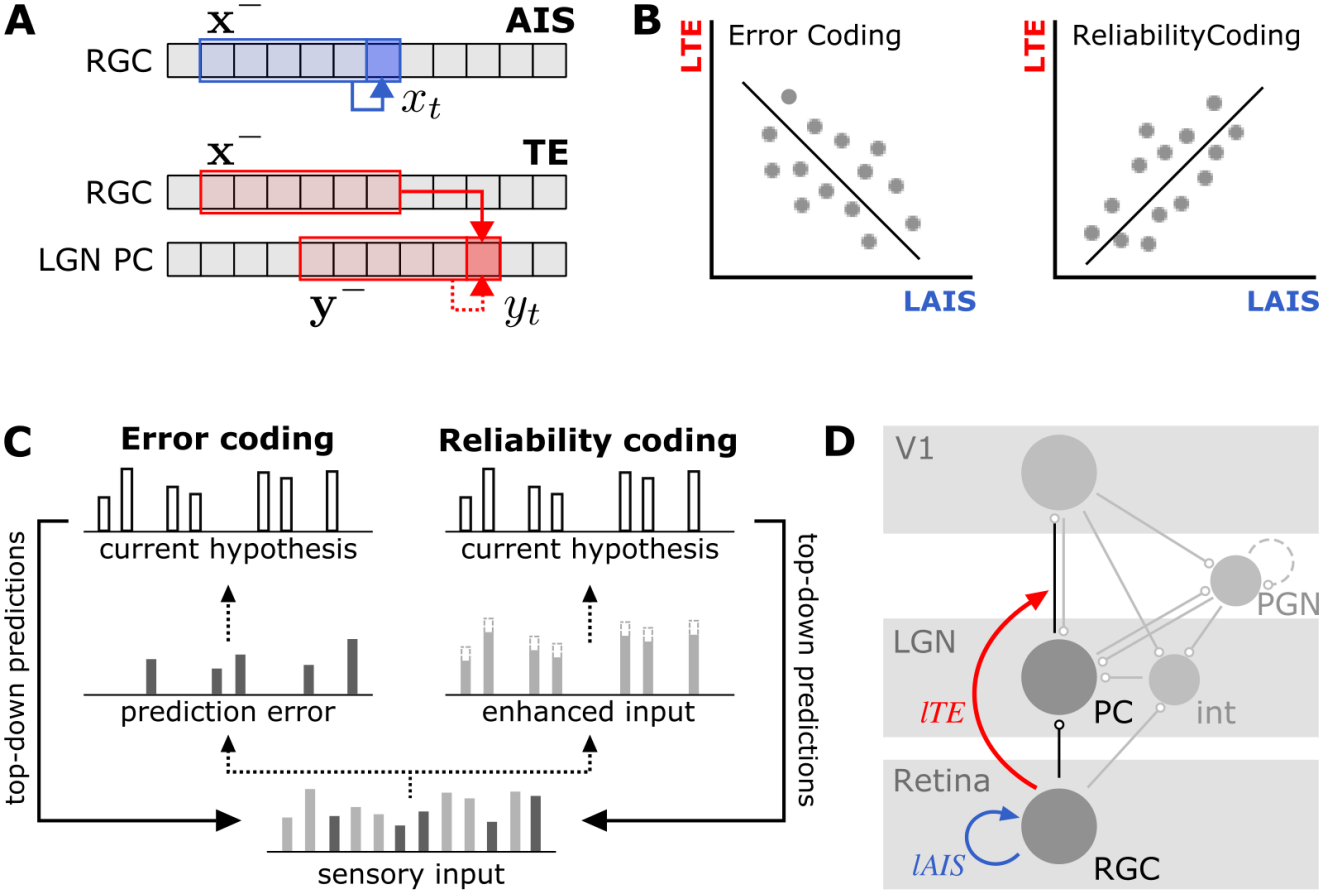
Overview of analysis approach. **A** Information-theoretic measures of predictability and information transfer: active information storage (*AIS*) quantifies the predictability of a processes’ current state *x*_*t*_ from its immediate past **x**^−^, transfer entropy (TE) quantifies the information transfer from a source process *X* to a target process *Y* by quantifying the predictability of the target’s current state, *y*_*t*_ from the sources’ past, **x**^−^, in the context of the target’s immediate past, **y**^−^. **B** Local storage-transfer correlations (LSTC) relating local AIS (*lAIS*) as a measures of predictability and local TE (*lTE*) as a measure of information transfer: if a neuron codes for predictable input a positive correlation is expected, if the neuron codes for unpredictable input, a negative correlation is expected (adapted from [5]). **C** Realizations of predictive coding in the cortex (adapted from [11]): bottom-up sensory input (dotted arrows) is compared to predictions propagated in top-down direction from a hierarchically higher cortical level (solid arrows) that represent the current prior about the input (white bars). Error coding assumes that bottom-up information represents predictions errors while reliability coding assumes that bottom-up information represents enhanced input. See main text for details. **D** Physiology of the retinogeniculate synapse and recording sites [12, 13]: Recordings were collected from in- and outputs to the synapse between retinal ganglion cells (RGC) and layer A principal cells (PC) in the lateral geniculate nucleus (LGN). We estimated local active information storage (*lAIS*, blue arrow) within the synapse input, and local transfer entropy (*lTE*, red arrow) between in- and output of the synapse. Schematic representations of known connections of PC and RGC are shown in grey (round markers indicate synapses): excitatory cells in layer 6 of primary visual cortex (V1) form feedback connections with LGN PC and also project to LGN inhibitory interneurons (int) and perigeniculate nucleus (PGN). Interneurons provide inhibitory input to LGN PC: intrageniculate interneurones (int) mediate feed-forward inhibition from RGC cells, while PGN cells provide recurrent inhibition [12, 14]; PGN interneurons further form reciprocal, inhibitory connections amongst each other (dashed line).

Using the local mutual information defined above, we can quantify how much of the information contained in a process (e.g., a neural signal) at the present moment *t* is predictable from its past. We assume that such a process denotes an ordered collection of random variables, **X** = {*X*_*t*_}, with realizations $x_{t}\in A_{X_{t}}$. We then quantify how predictable a single realization, *x*_*t*_, is from its past in the following way:

$$\begin{array}{c} \begin{array}{cc} lAIS(x_{t}:{\text{x}}^{-}) & \equiv i(x_{t}:{\text{x}}^{-})\\ & =\text{log}\;\frac{p(x_{t}|{\text{x}}^{-})}{p(x_{t})}\;, \end{array} \end{array}$$ (7)

where *lAIS* is shorthand for the local active information storage [10], and **x**^−^ is a realization of the (possibly infinite) past of the process up to time *t* (Fig 1A).

As already mentioned above the local mutual information forming the local active information storage need not be positive, i.e.:

$$\begin{array}{c} lAIS\left(x_{t}:{\text{x}}^{-}\right)<0\;\Leftrightarrow \;p\left(x_{t}|{\text{x}}^{-}\right)<p\left(x_{t}\right). \end{array}$$ (8)

This means that a negative *lAIS* indicates that the *x*_*t*_ that actually happened was less expected to happen, given the information in the past of the process, **x**^−^, than it was expected to happen without this information about the past. Put differently, the past **x**^−^ *mispredicted* the actual value of *x*_*t*_ by allocating the probability mass originally contained in *p*(*x*_*t*_) to other values in the conditional distribution. Since we assume that all probabilities, including the conditional probabilities above, are computed properly, this is a necessary misprediction, not one that could have been avoided. In other words, negative *lAIS* indicates unpredictable behavior of the process at time *t*. If we think of the overall process **X** as the input to a neuron then negative *lAIS* at time *t* means that the neuron must mispredict at *t*, based on the past of its inputs.

After defining predictability formally via *lAIS*, we next define how to measure whether the neuron is transferring mispredicted information onwards (as in coding for prediction errors) or whether it does not transfer information at those moments when the input is mispredicted (as in coding for the predictable input information only). A measurement of how much information is transferred by a neuron at each moment in time is given by the local transfer entropy [15].

### Measuring transmitted information as local transfer entropy from inputs to output

The information flowing from input process(es) (e.g., inputs to a neuron), **X**, to an output process, **Y**, (e.g., output of a neuron) at any moment in time, *t*, is given by the local transfer entropy [15] (Fig 1A):

$$\begin{array}{c} \begin{array}{cc} lTE({\text{x}}^{-}\to y_{t}) & \equiv i(y_{t}:{\text{x}}^{-}|{\text{y}}^{-})\\ & =\text{log}\;\frac{p(y_{t}|{\text{x}}^{-},{\text{y}}^{-})}{p(y_{t}|{\text{y}}^{-})}\;, \end{array} \end{array}$$ (9)

which quantifies the information transferred from the inputs’ past state, **x**^−^, about the present state in *Y*, *y*_*t*_, in the context of **Y**’s immediate past state, **y**^−^. Again, the *lTE* can be negative; in this case the negativity indicates that there is information in the output that is unexpected, given the past input from **X**,

$$\begin{array}{c} lTE\left({\text{x}}^{-}\to y_{t}\right)<0\;\Leftrightarrow \;p\left(y_{t}|{\text{x}}^{-},{\text{y}}^{-}\right)<p\left(y_{t}|{\text{y}}^{-}\right), \end{array}$$ (10)

i.e., negative *lTE* indicates that the *y*_*t*_ that actually happened was less expected to happen, given both the information in its own immediate past **y**^−^ and the past of the process **x**^−^ than without the information in **x**^−^. In other words, the past **x**^−^ mispredicted the actual value of *y*_*t*_ given the information obtained from **y**_*t*_. Similar to *lAIS*, this misprediction quantifies behavior of the process **Y** at time *t* that is unpredictable from the sources’ past, in the context of the target’s own past.

Using *lAIS* we can now quantify locally how predictable the current state of a single process is from its own past. Secondly, using *lTE* we can quantify locally, how much information is transferred from one process to a another. It should be mentioned already that the information-theoretic measures do not tell us something entirely removed from other descriptions of neural activity. Yet, their use allows for a rigorous, quantitative interpretation of biophysical observations in terms of neural information processing (see the Discussion section).

Next, we detail how relating both measures enables us to address the important issue of predictive coding.

### Local storage-transfer correlations (LSTCs) as an indicator for predictive processing

Using both *lAIS* on the input to a neuron and the *lTE* between its input and its output, we can now assess whether a neuron performs a predictive-coding like computation [5, 16] (Fig 1B and 1C). More specifically:

- If a neuron codes for the predictable parts of its input it should have a *highly positive* local information transfer *lTE* from input to output at moments *t* when the predictability of the input as measured by *lAIS* is *high*. That is, the correlation between *lTE* and *lAIS* should be *positive*.
- If, in contrast, a neuron codes for the unpredictable parts of its input, then local information transfer *lTE* should be *highly positive* at moments *t* when the predictability of the input as measured by *lAIS* is *very low* or even *negative*. That is, the correlation between *lTE* and *lAIS* should be *negative*.

The first variant of predictive coding theory has been proposed, for example, in adaptive resonance theory (ART) [17, 18] or the biased competition model [19, 20]. These theories assume that the signaling of bottom-up sensory evidence in the cortical hierarchy is facilitated for sensory input that matches top-down predictions by the current predictive model (Fig 1C). The second variant of theories has been proposed, for example, in [21–23], where it is suggested that bottom-up signals represent prediction errors, i.e., sensory input that is not predicted by the current internal model (Fig 1C). Here, top-down signals represent predictions made at a higher cortical area about the next lower area in order to “explain away” sensory input at the lower area [21–23]. The bottom-up error-signal then represents the part of the top-down prediction not explained away and thus signals the mismatch between prediction and input [24]. Both variants have been shown to be functionally equivalent such that an implementation of predictive coding can be achieved by both [25]. It is an ongoing debate which of the two proposed strategies neural systems use (see also [26–28], and the Discussion section).

Note that we here do not measure the predictions made by higher cortical areas directly, but use the *lAIS* of the input to the synapse to measure the predictability of the LGN’s input. We discuss the validity of this choice in detail in the Discussion (Section *Quantifying the self-predictability of neural signals as a proxy for predictions*). There, we also explain how the framework can be extended to accommodate additional neural signals that could reflect internal predictions explicitly made in higher cortical areas.

We want to highlight that the assessment of predictive coding using the above described local storage-transfer correlation (LSTC, Fig 1B) requires only minimal knowledge on the experiment that provided the data, namely, it is sufficient to know how to properly assess the probability distributions involved in the estimation of *lAIS* and *lTE*. The approach is thus applicable to data from a vast range of experiments, including those not specifically designed with predictive coding in mind. Most importantly, this approach does not require knowledge on what the brain or a neuron should predict.

### Partial information decomposition as a measure of state-dependent and -independent information transfer

Before we go on to describe how to estimate LSTC from data, we want to introduce the framework of partial information decomposition (PID) [7, 29–32] as a tool to investigate information transfer in neural processing in more detail. PID is a recent extension to classical information theory and has been widely applied in neuroscience (e.g., [5, 33–35]). PID allows to decompose information transfer measured by transfer entropy (TE) into contributions that are reflective of the calculation of a “generalized prediction error” versus contributions that indicate a relaying of predictable information. Such a decomposition can not be obtained using classical information-theoretic terms such as the (conditional) mutual information [7], hence, applying PID allows us to obtain additional evidence on whether transferred information serves the propagation of prediction errors or predictable sensory input that can not be obtained from mutual-information-based measures alone.

PID describes how two or more source variables provide information about a target variable, where each source may provide unique information (information that is only available from this particular source), redundant information (information that is available redundantly from two or more sources), and synergistic information (information that is only available when considering two or more input variables together) [7]. Note that in this study, we apply a non-localized measure of PID [36] (discussed in detail below), and therefore refer to averaged quantities only (for first proposals of localized PID measures see [31, 37]).

To illustrate how PID can be used to decompose TE [29], we first take a closer look at the calculation of the TE as the conditional mutual information *I*(*Y*_*t*_: **X**^−^|**Y**^−^). Here, conditioning on the target’s past state, **Y**^−^, influences the information the inputs’ past state, **X**^−^, provides about *Y*_*t*_ in one of the following manners,

- in the context of **Y**^−^, **X**^−^ may provide *less* information about *Y*_*t*_ such that *I*(*Y*_*t*_: **X**^−^|**Y**^−^) < *I*(*Y*_*t*_: **X**^−^);
- in the context of **Y**^−^, **X**^−^ may provide *more* information about *Y*_*t*_ such that *I*(*Y*_*t*_: **X**^−^|**Y**^−^) > *I*(*Y*_*t*_: **X**^−^);
- there may be no change in the information provided by **X**^−^ about *Y*_*t*_, such that *I*(*Y*_*t*_: **X**^−^|**Y**^−^) = *I*(*Y*_*t*_: **X**^−^), i.e., **Y**’s past is independent of **X**^−^ and *Y*_*t*_, and knowing **Y**’s past does not influence the information we obtain from **X**^−^ about *Y*_*t*_.

These changes in information contribution may be decomposed and quantified using PID terms [29]: The first case may be interpreted as scenarios in which information about *Y*_*t*_ is redundantly present in both past states, **X**^−^ and **Y**^−^, such that by conditioning on **Y**^−^ this redundant information is “removed” from the information **X**^−^ provides about *Y*_*t*_. The second case describes scenarios in which both past states provide synergistic information about *Y*_*t*_, which is “added” to the information **X**^−^ provides uniquely about *Y*_*t*_. Note that both redundant and synergistic information contribution can be simultaneously present in the interaction of two variables with respect to a third. The third case can be loosely thought of as the information **X**^−^ entering *Y*_*t*_ being both, independent of **Y**^−^ and being encoded into *Y*_*t*_ independently of **Y**^−^—thus, it reflects a unique information transfer from **X**^−^ to *Y*_*t*_. In sum, when calculating TE, i) we remove redundant information in **X**^−^ and **Y**^−^ about *Y*_*t*_, ii) we measure *synergistic* information jointly present in **X**^−^ and **Y**^−^ about *Y*_*t*_ [29], and iii) we measure the information provided *uniquely* by **X**^−^ about *Y*_*t*_.

### State-dependent transfer entropy as generalized prediction error

PID allows us to decompose information transfer from a cell’s inputs, **X**^−^ to its output *Y*_*t*_, conditional on the target’s past, **Y**^−^, into different contributions: We can quantify the information uniquely provided by **X**^−^ about *Y*_*t*_, independently of **Y**^−^, also termed *state-independent* TE [29], and we can quantify the information provided by **X**^−^ about *Y*_*t*_ synergistically with **Y**^−^, i.e., dependent on the state of **Y**^−^, also termed *state-dependent* TE [29]. One may think about the latter case as the target’s past state “decoding” the information transferred from the source to the target.

The computation of state-dependent TE, i.e. the synergistic part of the TE, is of particular relevance here, as the synergy reflects the computation of a “generalized prediction error” from the past state of the target cell (the prediction) and the past state of the input (the sensory evidence) and the error’s transfer by the target neuron. This can best be seen by considering that the computation of a binary error (e.g., in a spiking neuron) is analog to the XOR operation and that this operation leads to synergistic information: here, knowing only one input is not sufficient to know what the output of the system should be—this is only possible if both inputs are considered at once (see also the Discussion section).

### Partial information decomposition measures and estimation

The PID framework as introduced in [7] extends classical information theory to allow for the decomposition described in the last section and proposes an initial measure of the redundant information, from which all other PID terms can be calculated. However, more recent work has criticized this proposal for yielding unintuitive results in some cases. At the time of writing, finding an appropriate PID measure is still an active area of research and a series of alternative measures has been proposed [30–32, 36, 38, 39]. We here use a measure by Bertschinger et al. that is based on the unique information [36]. For a detailed discussion of PID, see for example [30].

An implementation for estimating Bertschinger et al.’s measure [36] has been proposed in [40, 41] and is available as part of the IDTxl toolbox [42].

### Estimation of information-theoretic quantities from data

Typically, in experimental neuroscience the probability distributions underlying observed data, which are necessary to calculate the quantities introduced above, are unknown and have to be estimated from data. We will therefore introduce the estimation of local information-theoretic quantities and PID terms from data in this section.

The most straightforward approach to estimating (conditional) mutual information from discrete data (Eqs 7 and 9) is by replacing probability mass functions by the relative frequencies of symbols observed in the data [43]. These so-called “plug-in estimators” are well-known to exhibit negative bias for finite data, for which analytic bias-correction procedures exist [44, 45]. These bias-correction approaches, formulated for non-local variants of mutual information, may be adapted for the use with localized measures to obtain locally bias-corrected estimators of *lAIS* and *lTE* (see supporting information S1 Text). Furthermore, statistical testing against estimates from surrogate data may be applied to handle estimator bias [46, 47] by treating the estimate as a test statistic compared against a Null-distribution generated from estimates from surrogate data.

Before applying estimators, past states of the time series involved have to be defined. In theory, both AIS and TE quantify the information contained in the semi-infinite past of a time series up to, but excluding, time point *t*. In practice, few observed systems actually retain information for an infinitely long time, such that most information is contained within the immediate past of the present system state, *X*_*t*_ [48, 49]. Hence, we can define an “embedding” of the time series, **X**^*S*^, i.e., a collection of past variables up to a maximum lag, selected such that the embedding is maximally informative about *X*_*t*_. In mathematical terms, we define the embedding, **X**^*S*^, such that the Markov property

$$\begin{array}{c} p\left(X_{t}|X^{S}\right)\approx p\left(X_{t}|X_{t-1},\ldots ,X_{0}\right), \end{array}$$ (11)

is fulfilled for all *X*_*t*_. In other words, *X*_*t*_ becomes conditionally independent of all variables prior to **X**^*S*^.

Several approaches for defining such an embedding exist. We here propose the use of a *nonuniform* embedding [50, 51], that selects variables from a set of past candidate variables, **C**, such that **X**^*S*^ becomes maximally informative about *X*_*t*_. A suitable algorithm that handles the computational complexity of selecting this set of variables is a greedy forward-selection strategy that maximizes the information contained in the variable set with respect to *X*_*t*_ using the conditional mutual information as selection criterion,

$$\begin{array}{c} C^{*}=\underset{C}{\text{arg}\;\text{max}}\;I\left(X_{t}:C|{\text{X}}_{i}^{S}\right)\;\forall C\in {\text{C}}_{i}, \end{array}$$ (12)

where ${\text{X}}_{i}^{S}$ is the set of variables already selected in the *i*th step of the algorithm and *C* are candidate variables from the set of candidates **C**_*i*_. The candidate set is defined as a collection of past variables up to time point *t*, **C** = {*X*_*t*−*l*_, *X*_*t*−*l*−1_, …, *X*_*t*−*k*_}, where *l* *k* denote a maximum and minimum lag with respect to *t*. Surrogate testing is used to evaluate whether the selected variable, C* provides additional information about *X*_*t*_ by testing whether $I(X_{t}:C^{*}|{\text{X}}_{i}^{S})$ is statistically significant. If so, C* is included into the embedding and removed from the set of candidates,

$$\begin{array}{c} {\text{C}}_{i+1}\leftarrow {\text{C}}_{i}\backslash C^{*}, \end{array}$$ (13)

$$\begin{array}{c} {\text{X}}_{i+1}^{S}\leftarrow {\text{X}}_{i}^{S}\cup C^{*}. \end{array}$$ (14)

For a detailed account of the estimation procedure including a hierarchical statistical testing scheme that handles the family-wise error rate of the repeated testing during the iterative candidate selection, see [52] and the implementation in [42].

The greedy strategy for constructing past states can be directly applied to find a non-uniform embedding of the past of a process **X**, such that we can estimate *lAIS* as

$$\begin{array}{c} lAIS\left(x_{t}:{\text{x}}^{-}\right)=i\left(x_{t}:{\text{x}}^{S}\right). \end{array}$$ (15)

For the construction of past states for *lTE* estimation, we first optimize the target embedding, **y**^*S*^ (which amounts to quantifying the active information storage in the target) [53], before optimizing the source’s embedding in the context of the target embedding,

$$\begin{array}{c} C^{*}=\underset{C}{\text{arg}\;\text{max}}\;I\left(Y_{t}:C|\{{\text{Y}}^{S},{\text{X}}_{i}^{S}\}\right)\;\forall C\in {\text{C}}_{i}, \end{array}$$ (16)

where sets **C**_*i*_ and ${\text{X}}_{i}^{S}$ are updated according to Eq 14. We can then estimate *lTE* as

$$\begin{array}{c} lTE\left({\text{x}}^{-}\to y_{t}\right)=i\left(y_{t}:{\text{x}}^{S}|{\text{y}}^{S}\right). \end{array}$$ (17)

By first optimizing the target’s past state, we make sure that we account for all information **Y**’s past provides about the current state *Y*_*t*_, before quantifying additional or novel information **X** provides about **Y**. This means that only information actually transferred between **X** and **Y** is taken into consideration when estimating *lTE*.

We used a software implementation of the proposed approach provided by the IDTxl Python toolbox [42], which internally makes use of plug-in estimators implemented as part of the JIDT toolbox [54]. For bias-correction, we used a Bayesian counting procedure implemented in the pyEntropy toolbox [55]. For estimation of PID measures, we used the measure by Bertschinger et al. [36] and an estimator by Makkeh et al. [40, 41], which is also part of the IDTxl toolbox. All analysis code has been made publicly available at https://github.com/pwollstadt/retinogeniculate_synapse/ [56].

### Empirical data set

We demonstrate the application of the proposed local information dynamics framework on spike train recordings from the retinogeniculate synapse of the cat. Spike trains were recorded from 17 retinal ganglion cells (RGCs) and monosynaptically coupled principal cells in the lateral geniculate nucleus (LGN) [8]. We estimated *lAIS* in the input to the synapse, i.e., the RGC spike train, and *lTE* between the input and the output of the synapse, i.e., from the RGC to the LGN spike train (Fig 1D). We calculated LSTC to test whether information was preferentially transferred whenever the input signal was predictable or when it was non-predictable. To support our analyses, we additionally used PID to decompose the information transferred into contributions reflecting the transfer of sensory input versus contributions reflecting the transfer of prediction errors.

A detailed description of surgical procedures, task, and data recordings can be found in [8].

#### Surgery

For electrode placement at the RGC and LGN (Fig 1D), adult cats of both sexes were initially anesthetized with ketamine (10 mg/kg, i.m.). For electrophysiological recordings, animals were placed in a stereotaxic apparatus and mechanically ventilated. Electrocardiogram (ECG), electroencephalogram (EEG), and expired CO_2_ were continuously monitored, while anesthesia was maintained with thiopental sodium (2 mg ⋅ kg^−1^ ⋅ h^−1^, i.v.). Thiopental administration was increased if physiological monitoring indicated a decrease in the level of anesthesia.

Once electrodes were positioned and minimum eye movement was ensured, the animal was paralyzed using vecuronium bromide (2 mg ⋅ kg^−1^ ⋅ h^−1^, i.v.). The pupils were dilated with 1% atropine sulfate and the nictitating membranes were retracted with 10% phenylephrine. Flurbiprofen sodium (1.5 mg/h) was administered to ensure pupillary dilation. The eyes were fitted with contact lenses and focused on a monitor located 1 m in front of the animal.

#### Visual task

Visual stimuli were created with a VSG 2/5 visual stimulus generator (Cambridge Research Systems) and presented on a gamma-calibrated Sony monitor with mean luminance of 35 cd/m^2^. Receptive fields were mapped using a binary white-noise stimulus that consisted of a 16 × 16 grid of squares [57]. Each square flickered independently between black and white according to an *m-sequence* [57, 58]. The monitor ran at a frame rate of 140 Hz. Approximately 4 to 16 squares of the stimulus overlapped the receptive field center of each neuron.

#### Electrophysiological recordings

Simultaneous single-unit recordings were conducted at the RGC and the contralateral layer A LGN cells. To maximize the chances that both cells were monosynaptically connected, a seven-channel multielectrode array (Thomas Recording) was placed in the LGN; through stimulation with a spot of light, the retinal area with the highest evoked response was identified. Cell responses were analyzed using an audio monitor.

Neural responses were amplified, filtered, and recorded with a Power 1401 data acquisition interface and the Spike 2 software package (Cambridge Electronic Design). The spikes of individual neurons were isolated using template matching, parametric clustering, and the presence of a refractory period in the auto-correlogram.

Recordings from 17 cell pairs entered further analysis. Recordings had an average length of 788.4 s (± 441.6 s SD, see supporting information S1 Table).

To assert connectivity between recorded cells, the cross-correlogram between both recordings was visually inspected for abrupt, short-latency peaks using a bin-size of 0.1 ms (see [8], Fig 1). The occurrence of such a peak was seen as evidence for a monosynaptic connection between RGC and LGN cell [59–61]. For peaks a baseline mean was calculated from bins 30 ms to 50 ms on either side of the peak bin. The peak bin and all neighboring bins with counts >3 SD were considered to contain retinal spikes triggering an LGN spike. The percentage of these spikes was termed the *efficacy* of the RGC [60–62]. Furthermore, an RGC’s *contribution* was defined as the percentage of LGN spikes that were triggered by a spike in the corresponding RGC. Contribution may be interpreted as the “strength of connection” between two cells in a pair [8]. We called an RGC spike *relayed* if it was followed by a LGN spike after its reconstructed information transfer delay, *u* (see next section).

For further analyses recorded spike trains were binned into 1 ms segments.

#### Estimation of lAIS and lTE from empirical data

We optimized nonuniform past-state embeddings for each cell pair recording using the greedy algorithm implemented in [42]. For *lAIS* estimation, we set the maximum lag, *j*, defining candidate variables for the embedding to 30 ms; for *lTE* estimation, we set the maximum lag in the source, *k*, to 40 ms and the maximum lag in the target, *l*, to 30 ms. These lags assume that only spikes with an inter-spike interval (ISI) of 30 ms and less are relevant for triggering a LGN spike [59, 60, 63–66], where especially ISIs <10 ms are effective in driving LGN responses.

For optimizing the *lTE* target past, we additionally accounted for a information-transfer delay, *u*, between RGC and LGN of up to 10 ms, which is in line with the cross-correlation observed between spiking in RGC and LGN [8]. We reconstructed *u* from the optimized embedding by identifying the lag of the past source variable that had the highest information contribution to the target’s current state, quantified by the conditional mutual information *I*(*x*_*u*_: *y*_*t*_|**x**^*S*^\*x*_*t*−*u*_)

$$\begin{array}{c} \hat{u}=\underset{u}{\text{arg}\;\text{max}}\;I\left(y_{t}:x_{t-u}|\{{\text{y}}^{S},{\text{x}}^{S}\backslash x_{t-u}\}\right)\;\forall x_{t-u}\in {\text{X}}^{s}. \end{array}$$ (18)

#### LSTCs calculation for empirical data

To investigate whether the retinogeniculate synapse preferentially transferred predictable or unpredictable information, we correlated sample-wise estimates of *lAIS* and *lTE* by calculating the Pearson correlation coefficient between both measures. Note that we may also calculate measures that capture relationships of higher order, e.g., the mutual information. However, since our goal was to infer whether the sign of the correlation was positive or negative, we calculated the linear correlation. Tests for statistical significance were performed using a permutation test with 1000 permutations.

## Results

### Optimization of estimation parameters

For estimation of local information-theoretic measures, we first optimized past states for *lAIS* and *lTE* estimation individually for each cell pair. Over all cell pairs, the mean lag of variables identified for the *lAIS* embedding was 7.63 ms (SD: 1.82 ms), and for *lTE* embedding was 2.75 ms (SD: 1.24 ms) for the source and 6.06 ms (SD: 1.53 ms) for the target embedding. The reconstructed delay, *u*, between the RGC and LGN cell was on average 2.81 ms (SD: 1.05 ms), while individual delays matched maxima in the cross-correlogram between RGC and LGN recordings. (Supporting information S1 Table provides descriptive statistics of data entering the analysis, and supporting information S2 Table lists all estimated parameters).

### LSTC

Based on the optimized past states, we estimated *lAIS* and *lTE* for all cell pairs and found significant storage and transfer in all pairs except for pair 5, which was excluded from all further analyses. For remaining cell pairs, we calculated the LSTC and found a significant, positive correlation coefficient for 14 of the remaining 16 pairs, indicating that local information transfer was higher at samples with higher local information storage (coefficients ranged from 0.0056 to 0.2675, see supporting information S3 Table for all correlation coefficients). With respect to predictive coding strategies, the positive LSTC indicates a higher transfer of information whenever an input sample was more predictable from its past, and less transfer when it was unpredictable. To demonstrate that also negative LSTCs are in principle possible, we show analyses for a simple toy example as supporting information S2 Text.

**Fig 2:**
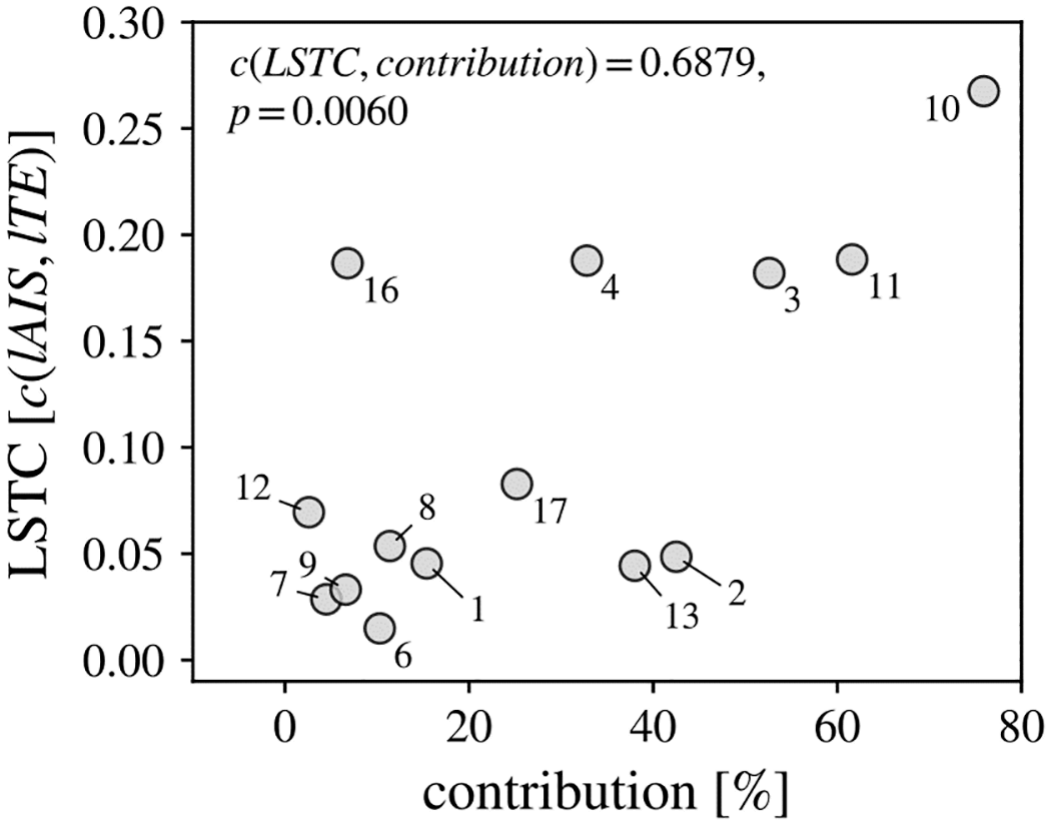
Correlation between contribution and local storage-transfer correlations (LSTC) for all spike pairs. LSTC was stronger for cell pairs with higher contribution (percentage of LGN spikes triggered by an RGC spike), i.e., synapses that were more strongly connected.

We further found that correlations were stronger in cell pairs with a high RGC *contribution* (Fig 2, *c*(*LSTC*, *contribution*) = 0.6879, *p* = 0.0030**). In a cell pair, the RGC’s contribution is defined as the percentage of spikes in the LGN cell that were triggered by a previous spike in the RGC and may be interpreted as the pair’s “strength of connection” [8]. Hence, the effect of predictable information being relayed across the retinogeniculate synapse was more pronounced in synapses that were more strongly connected. Estimates of the contribution of each cell pair were taken from [8].

### State-dependent and state-independent information transfer

**Fig 3:**
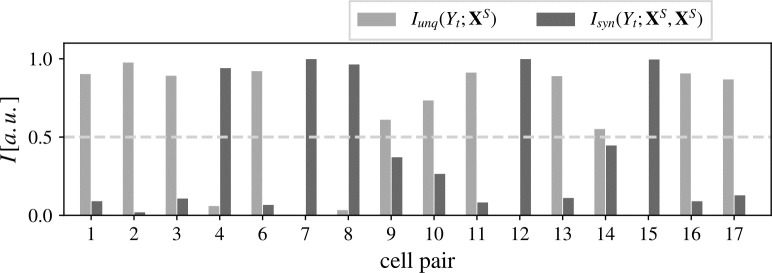
State-dependent and -independent information transfer from RGC to LGN cell. State-dependent and -independent information transfer from RGC to LGN cell, measured by the synergistic information, *I*_*syn*_(*Y*_*t*_; **X**^*S*^, **Y**^*S*^) (dark gray), and unique information, *I*_*unq*_(*Y*_*t*_; **X**^*S*^) (light gray). In 11 out of 16 pairs, more than half of the transferred information was independent of the LGN cell’s past state.

Additional to LSTCs, we estimated unique information in the RGC past, *I*_*unq*_(*Y*_*t*_: **X**^*S*^), and synergistic information in the RGC and LGN pasts, *I*_*syn*_(*Y*_*t*_; **X**^*S*^, **Y**^*S*^), about the spiking behavior of the LGN principal cell [36, 40] (Fig 3). In 11 out of 16 cell pairs with significant information transfer, the unique information provided by the RGC’s past state, **X**^*S*^, dominated the information transfer from RGC to LGN. Hence, information transfer was governed by information transfer independent of the state of the LGN’s past state. Again, this supports the notion of information transferred mainly in a bottom-up fashion, i.e., transfer independent of the target cell’s state. There was no correlation between the magnitude of the unique or synergistic information and the LSTC.

### Information dynamics of relayed and non-relayed RGC spikes

The remaining results relate the estimated *lAIS* and *lTE* to spiking statistics of the raw spike trains. We performed these analyses to demonstrate that our results are plausible given the biophysiological mechanisms underlying the activity at the retinogeniculate synapse.

**Fig 4:**
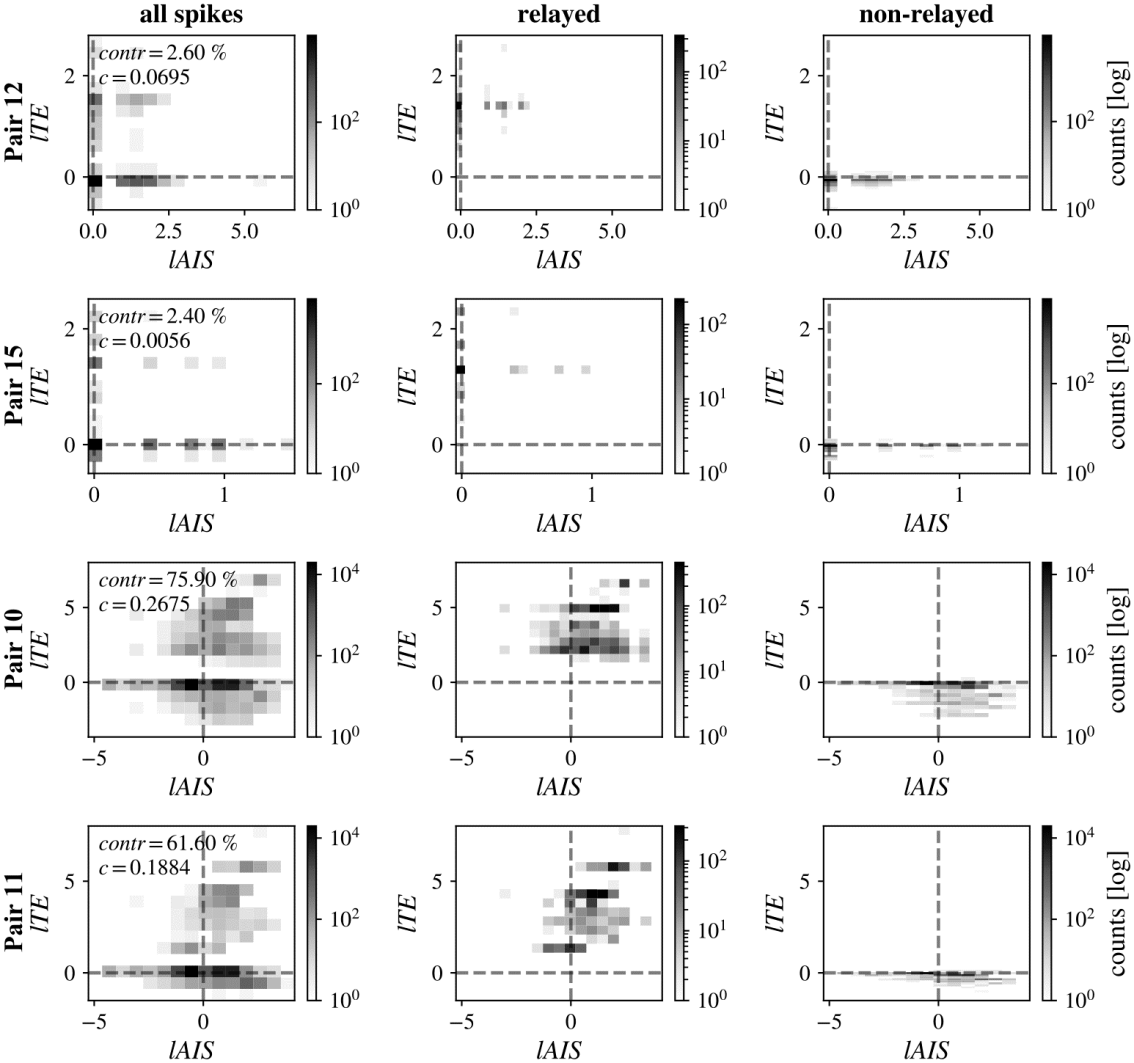
Local storage-transfer correlations (LSTC) for exemplary cell pairs. Histograms of LSTC for representative cell pairs with highest (pairs 10 and 11) and lowest (pairs 12 and 15) contribution, respectively. The first column shows histograms for all spikes, the second column for relayed spikes, and the third column for non-relayed spikes. An RGC spike was considered relayed to the LGN if it was followed by an LGN spike with the delay reconstructed during *lTE* estimation. Rows show individual cell pairs. Relayed spikes showed positive *lTE* and generally positive *lAIS*, while non-relayed spikes led to zero or negative *lTE* and lower *lAIS*.

First, we investigated whether *relayed* RGC spikes differed in their local information dynamics from *non-relayed* spikes. An RGC spike was considered a relayed spike if it was followed by an LGN spike with the reconstructed information transfer delay, *u*, while all other RGC spikes were considered *non-relayed*. For all analyzed pairs, only a fraction of spikes was relayed. Fig 4 shows two-dimensional histograms of *lAIS* and *lTE* values for four representative cell pairs. Histograms are shown for all spikes, as well as only for relayed and non-relayed spikes, respectively.

**Fig 5:**
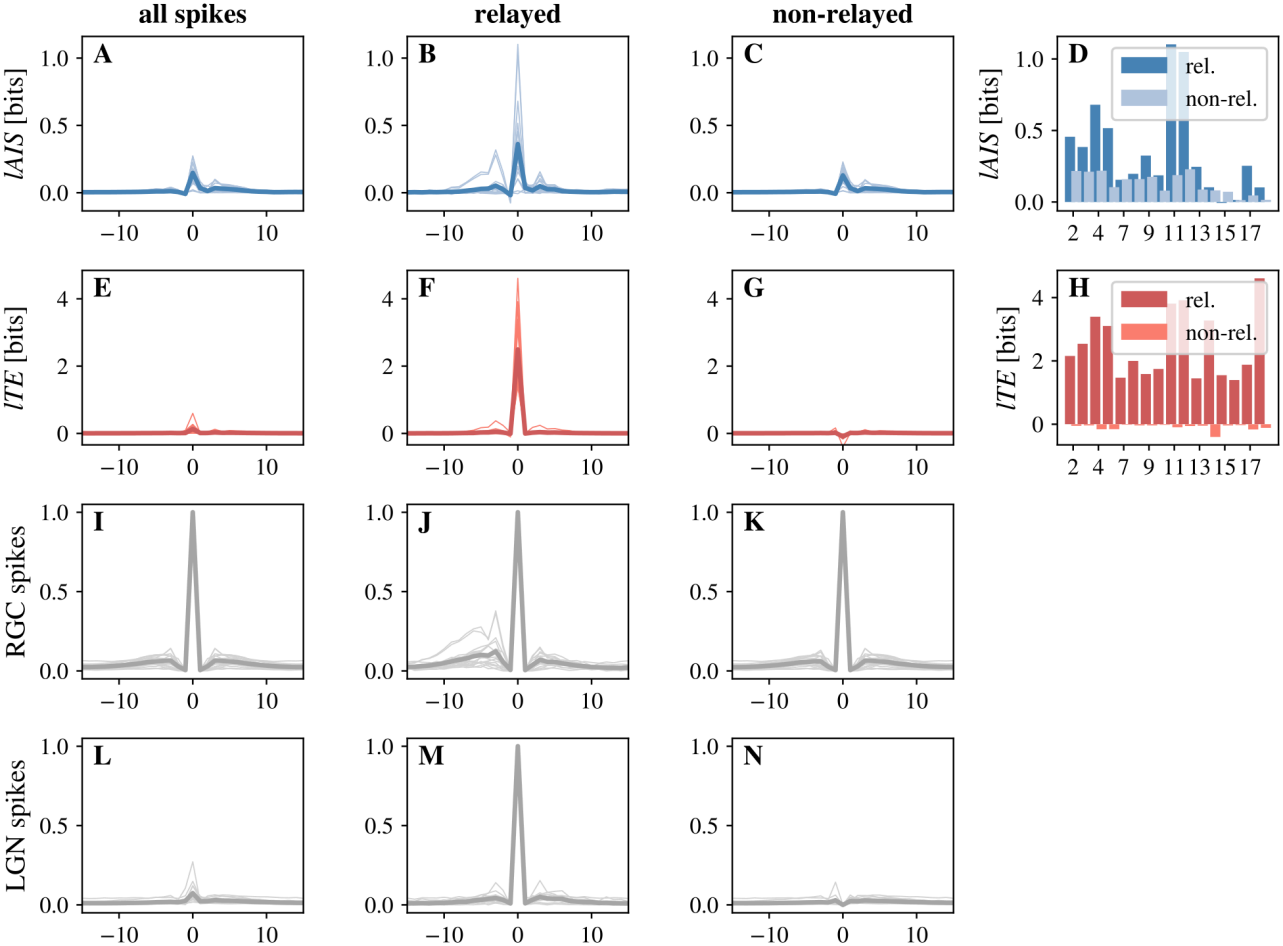
Information dynamics of relayed versus non-relayed RGC spikes. An RGC spike was considered relayed to the LGN if it was followed by an LGN spike with the delay reconstructed during *lTE* estimation. Relayed spikes were accompanied by higher *lAIS* than non-relayed spikes. Also, relayed spikes led to high *lTE* in comparison to non-relayed spikes. **A–C** Spike-triggered average (STA) for *lAIS* values (**A** all RGC spikes, **B** relayed spikes, and **C** non-relayed spikes); **D** Mean *lAIS* for relayed and non-relayed RGC spikes, for each cell pair. *lAIS* was higher for relayed (dark blue) than for non-relayed (light blue) RGC spikes (*p* < 0.001*** for a permutation test with 1000 permutations). **E–G** STA for *lTE* values (**E** all RGC spikes, **F** relayed spikes, and **G** non-relayed spikes); **H** Mean *lTE* values for relayed and non-relayed RGC spikes, for each cell pair. *lTE* was higher for relayed (dark red) then for non-relayed (light red) RGC spikes (*p* < 0.001*** for a permutation test with 1000 permutations). **I–K** STA of RGC spike trains (**I** all RGC spikes, **J** relayed spikes, and **K** non-relayed spikes). Relayed RGS spikes were more often preceded by a spike than non-relayed spikes. **L–N** STA of LGN spike trains, aligned with corresponding RGC spike train with respect to the reconstructed delay (**L** all RGC spikes, **M** relayed spikes, and **N** non-relayed spikes). As expected, relayed RGC spikes were always followed by an LGN spike, while this was not generally the case for all spikes.

On average, relayed RGC spikes were accompanied by higher *lAIS* and *lTE* compared to non-relayed spikes (Figs 4 and 5). Results indicate that, first, relayed spikes were in general more predictable from the RGC’s cells immediate past spiking behavior. Second, relayed spikes were accompanied by higher local information transfer, while non-relayed spikes were accompanied by negative local information transfer. Negative *lTE* here means that for some cell pairs, in the absence of an LGN spike, the RGC’s state (spike) was misinformative about the next state of the LGN (no spike). In other words, observing a prior RGC spike lowered the probability of observing no spike in the LGN.

Relayed spikes were characterized by both higher *lAIS* and *lTE* values. We were able to classify whether a spike was relayed from its *lAIS* value above chance, using a k-nearest neighbor classifier with *k* = 5 (classification accuracy was also higher than the baseline model, see supporting information S4 Table). However, note that *lAIS* may be seen as a different representation of spiking statistics of the RGC, i.e., the number of spikes and ISI in a given time window (as can be seen, for example, when considering spike-triggered averages of *lAIS* and RGC spike counts in Fig 5). As a result, whether a spike was relayed could be equally well predicted from the spike count of all spikes up to 30 ms prior to an RGC spike, or the RGC spike’s prior ISI (see supporting information S4 Table). We therefore want to highlight that the estimation of *lAIS* provides no additional, mechanistic explanation on *when* a spike is relayed at the retinogeniculate synapse—it rather provides a computational interpretation of the mechanisms already known (see also the Discussion section).

### Information dynamics of inter-spike intervals

**Fig 6:**
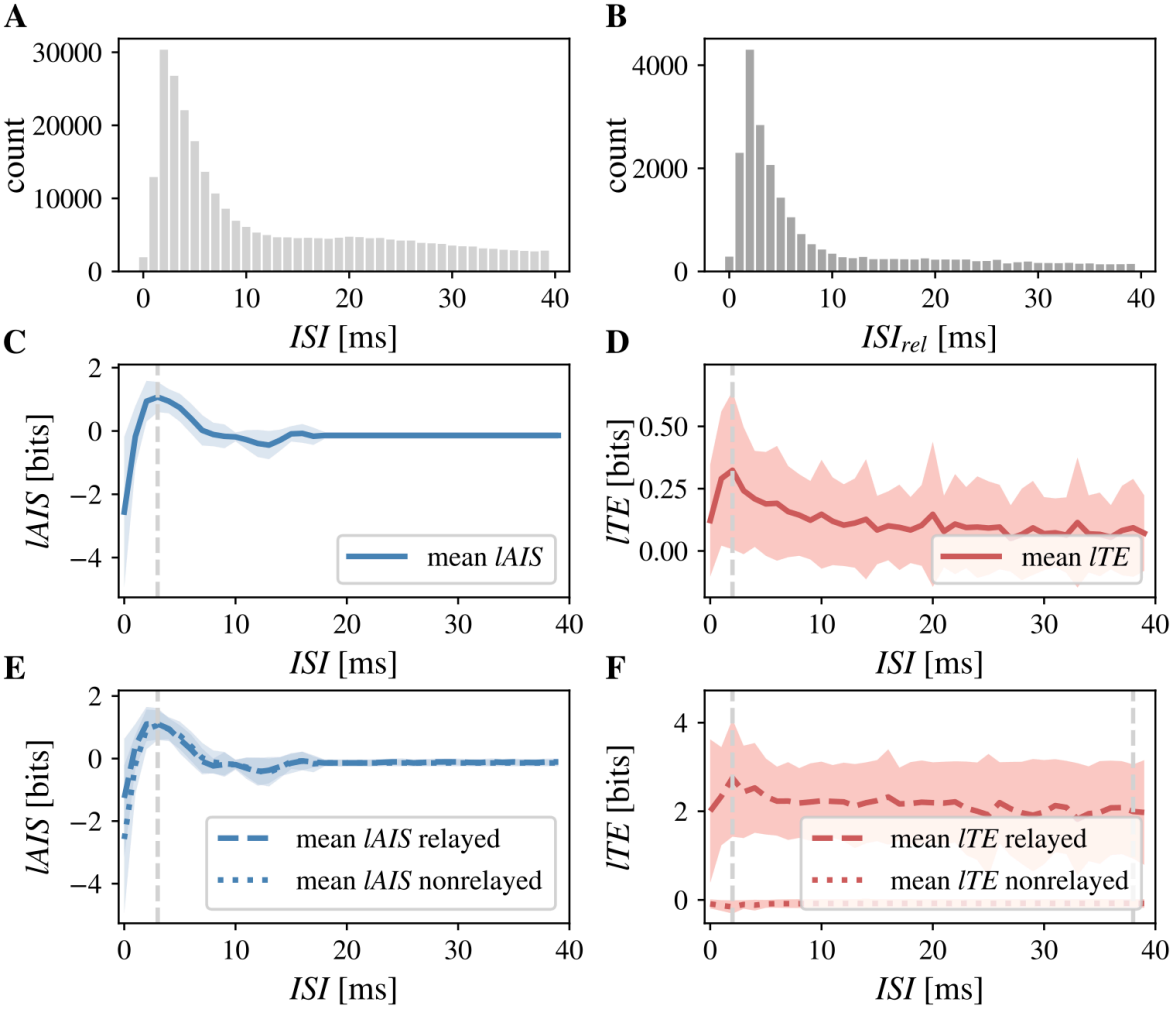
Information dynamics of inter-spike intervals (ISI). Predictability of the RGC spike train, measured by *lAIS*, was highest for spikes with the most frequent preceding ISI. Information transfer across the synapse, measured by *lTE*, was also highest for RGC spikes with the most frequent preceding ISI. While predictability was high for relayed and non-relayed RGC spikes, information transfer was only high for relayed spikes. **A** Distribution of ISI pooled over all cell pairs (maximum at 2 ms). **B** Distribution of ISI for relayed RGC spikes, pooled over all cell pairs (maximum at 2 ms). **C** Mean *lAIS* at RGC spike as a function of the preceding ISI (maximum at ISI = 3 ms, dashed vertical line, shaded area indicates ±1*SD*); **D** Mean *lTE* at RGC spike by preceding ISI (maximum at ISI = 2 ms, grey vertical line, shaded area indicates ±1*SD*); **E** Mean *lAIS* at relayed (dotted line) and non-relayed (dashed line) RGC spikes as functions of the preceding ISI (maxima at ISI = 3 ms for relayed spikes, dotted vertical line, and at ISI = 3 ms for non-relayed spikes, dashed vertical line, shaded area indicates ±1*SD*); **F** Mean *lTE* at relayed (dotted line) and non-relayed (dashed line) RGC spikes as functions of the preceding ISI (maxima at ISI = 2 ms for relayed spikes, dotted vertical line, and at ISI = 38 ms for non-relayed spikes, dashed vertical line, shaded area indicates ±1*SD*).

Last, we investigated the local information dynamics of RGC and LGN spikes as a function of the preceding ISI, as ISIs have been reported to have an effect on whether an RGC spike drives a response in the corresponding LGN cell [59, 60, 63–66]. We calculated ISIs by subtracting the spiking times of all consecutive spikes in the RGC spike train (Fig 6A).

Average *lAIS* was positive for RGC spikes with a preceding ISI of 2 ms to 7 ms, with a maximum at 3 ms. The *lAIS* was negative for all other investigated ISIs (Fig 6B). Hence, the most frequent ISIs lead to higher predictability of the spike. When differentiating between relayed and non-relayed RGC spikes, *lAIS* was positive for ISI of 1 ms to 6 ms for relayed spikes while the range of positive *lAIS* values for non-relayed spikes was 2 ms to 7 ms. Overall, relayed and non-relayed spikes did not differ in *lAIS* as a function of ISI (Fig 6D). *lTE* was positive over the whole range of investigated ISIs (Fig 6C). However, when differentiating between relayed and non-relayed spikes, *lTE* was negative on average for all ISIs for non-relayed spikes with a minimum at 2 ms (Fig 6E).

We further investigated RGC spike *tuples*, because whether RGC spikes are relayed is mostly influenced by the most recent previous spike while events further in the past have only minor influence [60]. Tuples are defined as two spikes with an ISI below a given threshold and a “silence time” preceding the first spike to ensure a comparable level of prior activity [60]. We here used a silence time and maximum ISI of 20 ms, which covers the maximum history length used in the estimation of *lAIS* and *lTE* (supporting information S2 Table), such that spikes with a prior ISI >20 ms did not influence *lAIS* and *lTE* estimates.

**Fig 7:**
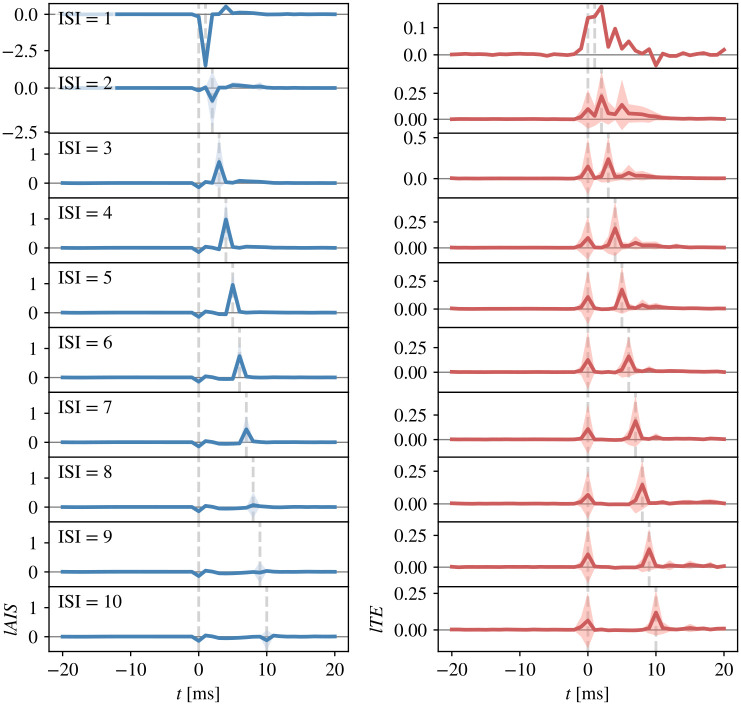
Spike-triggered averages (STAs) for spike tuples. STAs for spike tuples with a silence time of 20 ms and inter-spike interval (ISI) up to 20 ms (aligned on first spike in a tuple). Left column shows *lAIS* values averaged over cell pairs for ISI of 1 ms to 10 ms, right column shows averaged *lTE* values (shaded areas indicate ±1*SD*). The *lTE* values are shifted by the individual delay between RGC and LGN cell for each pair such that a spike at index *t* = 0 indicates a transferred spike with a delay corresponding to the reconstructed information transfer delay. The predictability of the second RGC spike in a tuple, measured by the *lAIS*, was high for ISI from 3 ms to 7 ms, while first spikes in a tuple were unpredictable as indicated by no or negative *lAIS*. Information transfer measured by *lTE* was high for all RGC spikes with highest values for the second spike in a tuple.

We computed spike triggered averages (STA) of *lAIS* and *lTE* values for spike tuples (two consecutive spikes with an ISI <20 ms, Fig 7). On average, the first spike in a tuple was associated with negative *lAIS*, indicating that the spike’s immediate past, i.e., the silence time, was misinformative about the spike. For an ISI of 3 ms to 7 ms, the second spike was associated with increased *lAIS*, relative to the average *lAIS* in the silence time, indicating high predictability from the immediate past. On average, *lTE* values were slightly increased for the first and second spike in a tuple, with higher values for the second spike. In sum, the predictability of an RGC spike strongly dependent on prior spiking activity, with higher predictability if the ISI was between 3 ms and 8 ms.

## Discussion

We introduced an information-theoretic framework for testing predictive coding strategies in neural data. The framework expresses predictive coding concepts, namely predictability, predictions, and prediction errors, in terms of information-theoretic quantities, which can be immediately estimated from data. Hence, the framework does not rely on markers of predictive coding that have to be defined a priori, but on properties of the data itself. As a result, the framework enables the investigation of neural processing strategies in any data set, independently of the experimental task under which the data were collected. We applied the framework to spike recordings from the retinogeniculate synapse of the cat and identified the preferred coding strategy of the synapse, namely, the transfer of predictable over unpredictable input.

### Evidence for coding for predictable input found at the retinogeniculate synapse

We applied the proposed local information dynamics framework to investigate which of two alternative predictive coding strategies were used at the retinogeniculate synapse. In particular, we tested, whether the synapse coded for unpredictable or surprising input and transferred prediction errors (e.g., [21–24]), versus the synapse coded for predictable input and transferred sensory input matching top-down information (e.g., [17, 18] or [19, 20]). Currently, it is an area of active research which of the two proposed strategies neural systems use. Both strategies have been shown to be equivalent on a *functional level* (they use the principle of predictive coding to realize perception and action in the cortex), while they differ on an *algorithmic level* [67], and as a consequence in their implementation. Spratling and colleagues showed that both strategies are equivalent in their ability to realize predictive-coding-like information processing in artificial neural networks [25]. This was supported by Kveraga et al. [26], who suggest that different realizations of predictive coding theory could be easily accommodated by a computational model of top-down and bottom-up information processing presented in [27] (see also [28] for a further comparison of theories on top-down activity).

Here, our framework presents a novel way to test which processing strategies are used by neural systems. Applying the proposed framework, we found that the retinogeniculate synapse preferentially coded for predictable input. In 15 of 17 investigated cells, local predictability of the input correlated positively with local information transfer between input and output indicating the preferential transfer of predictable input. Also, we found that RGC spikes were more efficient in driving an LGN response if they were highly predictable from their immediate past. Lastly, using PID [7, 36], we showed that predominantly unique information in the RGC was transferred in most of the investigated cell pairs, which indicates that primarily sensory evidence rather than evidence of prediction errors was transferred across the synapse (discussed in detail in Section *Quantifying prediction errors in neural signals*).

### Local information dynamics as a semantics-free approach to investigating neural computation

#### Avoiding circular arguments in the investigation of predictive coding theory

We propose a framework that is applicable to data collected also from experiments not specifically tailored to investigate predictive coding. One key motivation for such a framework was to avoid the use of circular arguments where the researcher’s assumptions on what inputs the brain should predict are used to design stimuli and paradigms, that are then used in neurophysiological experiments to test whether and how the brain predicts these inputs (compare, for example, previous studies investigating the facilitation of predicted input over the propagation of prediction errors [19, 68–70]). As a result, the experimenter’s interpretation of neural activity becomes dependent on the a priori defined theory motivating the experimental setup and the expected manifestation of a prediction error in the data (see also [4, 71–73]). This motivation is based, not least, on the difficulties we experienced ourselves when designing and performing predictive coding experiments, and interpreting the data (e.g. in [74]). However, we do not want to suggest that all experiments necessarily suffer from these difficulties. Rather, we fully acknowledge that in some cases the necessary knowledge to carefully design non-circular predictive coding experiments will be available. However, we are concerned that this is not the case in general.

The problem of “interpreting” neural activity in terms of predictions or prediction errors becomes even more severe if arbitrary processing elements in the cortex are investigated in isolation, e.g., single cells, whose computations, as well as input and output are far removed from any human-understandable function (i.e. we have to treat it as “intrinsic computation” in the sense of [75]). Here, an approach is required that analyzes signals not from an “experimenter-as-receiver”, but a “cortex-as-receiver” or “neuron-as-receiver” point of view [76]. The former view assumes that neural signals at arbitrary processing stages carry human-understandable information—which may express a misleading view on information processing in the brain in general—, while the latter view considers the question of how other processing units in the cortex view available information.

Here, local information dynamics allow the investigation of computations performed by arbitrary processing units as they allow to immediately express information processing concepts without relying on the interpretation of the recorded data in terms of the experimental task performed. Instead, the concepts expressed become measurable properties of the data and allow to formulate testable hypotheses about competing processing strategies. In sum, the presented approach allows for a straightforward testing information-processing strategies in arbitrary neural systems, and opens up the possibility of testing predictive coding theories on data from a myriad of neurophysiological experiments not initially designed with predictive coding in mind. In the following we will discuss the formulation of predictive coding concepts in terms of information-theoretic quantities in more detail.

#### Quantifying the self-predictability of neural signals as a proxy for predictions

We quantified the *self-predictability* of the input signal to the synapse in order to quantify what portion of the signal was predictable from its own immediate past. We used this predictability as a proxy for measuring and quantifying an actual prediction of the synapse’s input from all available inputs (e.g., through feedback connections from the cortex to the LGN principal cell, etc.).

As the (local) AIS is a relatively novel concept in the analysis of neural information processing, a few words on its interpretation and validity in a biological context are necessary.

We would like to start by helping the reader to understand how this analysis is unaware of what part of the external world is being encoded in the neural spike train—and why this approach is nevertheless useful. As our approach only considers neural signals, and not the state of the outside world that caused them, concerns may arise for example about losing the distinction between task-relevant information encoded in spikes and task-irrelevant “noise spikes”, and also about losing information on the relevance of the inputs for the organism as a whole. However, this information is also not available directly to the neurons under study—it only becomes available to these neurons if it is provided via additional neural signalling—which we can indeed take into account in our approach. In other words, our approach can in principle consider information about signal versus noise, relevance, and other aspects of outside-world information, as long as this information is actually made available to the neurons under study by signalling from other neurons. (In the present experiment most additional influences, e.g. from cortical feedback, will possibly be negligible due to the anesthetized state of the animal, and the random nature of the stimulus.) This generality of our approach may be potentially overlooked as our proof-of-principle analysis focused on a single (dominant) input spike train. However, additional neural sources *N* of information useful for the prediction of a neural signal *S*, such as feedback from higher cortical areas, can be easily incorporated into the definition of the predictable information in *S* via considering the joint mutual information, defining the predictable information as *pAIS*:

$$\begin{array}{c} pAIS=i(S^{+}:S^{-},N^{-}), \end{array}$$ (19)

reflecting the predictable information in *S*^+^ provided by both, the past of the additional neural information sources *N*, *N*^−^, and the past state of *S*, *S*^−^, jointly. A specific application of *pAIS* is discussed in Section *Potential influences of anesthesia on cortical feedback and predictive coding*, below.

With respect to interpreting the predictability implied in our approach, it is also important to consider which potential sources of predictability exist and in what respect the origin of predictability matters. In the neural spike train itself, there are in general two possible sources of predictability: first, predictability arising from temporal statistical dependencies in the input to the organism, and second, internally generated temporal dependencies arising somewhere along the pathway to the receiving neuron of interest. Does one of these matter more than the other? To answer this question it makes sense to take the point of view of this neuron receiving the spike train. This neuron, throughout its existence has received nothing but the incoming spikes, and has no access to any “ground-truth” about the outside world, and temporal regularities in this outside world. So from a neural information processing perspective, the above distinction must vanish. Thus, for the analysis framework presented here, it does in principle not matter whether the stimulus input to the retina was predictable or not; all that matters is whether the incoming spike train from the retina was predictable at the level of the LGN. So from a neuron-centric perspective an analysis without the stimulus properties seems to us to reflect the circumstances a neuron finds itself in.

Nevertheless, we acknowledge that the proof-of-principle analysis presented here is an extreme case of application: The random stimuli lack all predictability, except for the short life-time of a video-frame. Thus, any predictability in the spike time series is internally generated in the retina. As a consequence, one may ask whether our analysis—although technically sound—is truly relevant, and whether the example analyzed here supports a more general applicability. For two reasons we think this is indeed the case—first, AIS must certainly further rise in all processing stages close to a predictable stimulus—compared to the unpredictable one used here, thus increasing the signal-to-noise ratio relevant for its estimation; second for a neuron receiving a spike train, it will essentially be difficult to distinguish internal from external sources of predictability in that spike train—at least without resorting to information from other (neural) channels.

A last important point is that using the AIS as a proxy for the predictability of an input signal introduces two more approximations: The first approximation is that AIS as a mutual information is used in place of an actual predictive model for the RGC inputs embodied in the organism. This mutual information is an upper bound on the mutual information between any model prediction based on the RGC inputs and the actual future samples of a time series. This holds under the further assumption that we choose the input’s past state for the estimation of AIS such that we indeed capture all relevant statistical regularities. In theory, the AIS is defined as the mutual information between a processes’ present state and its *semi-infinite* past [10]. Hence, in practice one has to find a suitable, finite embedding that covers only the *relevant* past [10, 49, 50]. Such an embedding is found through the approach used here, where we optimized a non-uniform embedding that covered a time horizon of 30 ms, which was identified in previous work as the time horizon over which spikes affect future spiking behavior of the RGC [59, 60, 63–66].

The second approximation introduced in our analyses relates to the practical estimation of the *lAIS* from spike trains. With limited data, this can lead to biased results, either by overestimation for the case of severely limited data availability or by underestimation if the analyzed history length is artificially shortened to curb “curse of dimensionality” problems in the estimation (see also [77]). Given the large amount of data available here we do not consider this limitation to apply.

#### Quantifying prediction errors in neural signals

We can use predictability not only as a proxy for the prediction of an input signal, but also as a proxy for *inevitable* prediction errors: If the input’s predictability is low for certain events, and thereby its *lAIS*,—according to our first assumption above—any reasonable model predicting the synapse’s next state from this input must generate a prediction error—simply by virtue of reflecting the underlying probabilities.

As a second approach to quantifying prediction errors, we proposed to calculate the synergistic portion of the information transfer between the synapse’s in- and output, using the recently proposed PID framework. In particular, we propose that high synergistic information between the RGC’s past state and the LGN cell’s past state about the LGN cell’s next state reflects transmission of a prediction error. This is because if the target cell’s next state reflects a prediction error, both inputs must be known to compute the output as they contribute to the computation of that next state: the past state of the input cell, providing the sensory evidence, and the past state of the target cell, providing the prediction. The error would then be computed from comparing the two inputs, leading to a response if there was a mismatch between the two states. Technically, for single spiking events, perfectly determining the occurrence of a prediction error is equivalent to a binary XOR operation, which leads to purely synergistic information between the two inputs and the output, according to PID theory (see e.g. [78] on the exact definition of synergy). While it is also well known that single neurons can only approximate a binary XOR, this would still lead to considerable synergistic information. Conversely, if the information transfer across the synapse served the propagation of predictable information, we would expect low synergistic information and some unique information in the input about the output (in a process similar to a binary AND operation). The latter scenario is in line with our empirical findings, indicating that the input to the synapse provided unique information about the next state of the target cell (observing a spike in one source increases the probability of observing a spike in the output).

#### Quantifying information transfer between neural signals

We estimated information transfer across the synapse using TE, which serves as a natural measure of information transfer serving predictive coding, because it quantifies the transfer of *novel* information from input to output of the receiving cell. As discussed in the previous section, the measured transfer considers both information uniquely provided by the synapse’s input, and information transferred due to synergistic effects between the input’s and the target cell’s past states.

Next, we will review how our information-theoretic results relate to possible biophysical implementations of the computations performed.

### Linking information-theoretic results to biophysiology

#### Biophysiological mechanisms underlying LSTCs

We found that information transfer was highest for highly predictable RGC spikes and that these input spikes were typically preceded by another input spike with a short advance. Our findings are in line with previous studies showing that RGC spikes with a preceding spike were more effective in driving an LGN response than single spikes [8, 60, 64, 66, 79], and that this efficacy was even higher for ISI <10 ms [60, 64–66, 79–81].

It has been hypothesized that double spikes are important to enable temporal summation at the post-synaptic membrane: Carandini and colleagues [82] presented a model of the retinogeniculate synapse in which information transfer was governed by temporal summation of pre-synaptic excitatory postsynaptic potentials (EPSP). Here, EPSPs remained approximately constant or even increased for smaller ISI. Hence, the dominant biophysical mechanism enabling information transfer at the synapse seemed to be post-synaptical summation rather than a change in pre-synaptic conditions due to enhanced spike-rates. This fits with the LGN’s limited ability to integrate spikes over large time windows, where the typical time constant for X- and Y-cells in the LGN is measured to be 15 ms to 22 ms [83]. The cells’ true ability to perform temporal summation may be even lower because the time constant may not be a suitable measure of the ability for temporal integration under real-world conditions [84]. Temporal summation as a mechanism is further supported by the fact that LGN principal cells receive input from just a small number of RGCs of which one is typically the main driver [65, 85]. Also, single RGC cells are able to drive the target LGN principal cell [60, 63, 64, 66, 80], such that population coding is an unlikely mechanism for the information transfer at the retinogeniculate synapse. Last—as was noted by Rowe and colleagues—contribution rises strongly under “structured” visual stimulation [64].

In sum, temporal summation over incoming spiking activity on short time-scales is a likely mechanism for driving information transfer from RGC to LGN principal cells. Our findings are compatible with this mechanism, as information transfer was highest for the second RGC spike in tuples with short ISI. This second spike also was highly predictable from past activity, explaining the observed LSTCs based on the above biophysical mechanism. Furthermore, we found predominantly unique information transfer from RGC to LGN, which is in line with the fact that almost all LGN spikes are triggered by an RGC spike [8, 80].

We conclude that our proposed framework yields results that have a plausible mechanistic explanation on the biophysiological level—yet, the framework adds an explanatory layer to the mere biophysiological description by casting information contained in the synapse’s spiking statistics into a quantitative and human-interpretable form. Indeed, our finding of a preferential transfer of predictable input sheds an interesting light on the findings in [8]: predictable input to the LGN cells (spike tuples) is produced when an RGC cell is stimulated by its preferred input. Thus, the signals relayed by LGN cells are strongly representational in nature, rather than differential.

#### Potential influences of anesthesia on cortical feedback and predictive coding

In our analysis, we used recordings from animals under anesthesia, which may affect our results due to the well-known change in information transfer under anesthesia, predominantly in top-down direction [86–91]. Under anesthesia top-down information transfer from V1 to the LGN is very likely reduced. V1 affects LGN function via direct and indirect connections (e.g. [12, 14, 92]), whose functional role may vary between facilitation and suppression of LGN spiking [92, 93]. As a result, the algorithm embodied by the retinogeniculate synapse may change if the cortex is active during recordings.

However, investigating information processing at the retinogeniculate synapse while V1 is active would allow us to integrate recordings from V1 as second input to the LGN into our analysis. This approach is an alternative to quantifying prediction errors by measuring information storage in the RGC input alone, circumventing the limitations discussed above. As laid out above in Eq 19 our framework is easily extendable to include additional sources. For our test case such additional sources of information could for example come from area V1. If V1 signals were available these could be included by defining the *predictable* information that the RGC and V1 jointly provide about the RGC input spike train to the LGN as:

$$\begin{array}{c} pAIS=i(RGC^{+}:RGC^{-},V1^{-}). \end{array}$$ (20)

Similarly, the PID-based analysis of predictive coding strategies can be adapted: if the synapse coded for prediction errors, we would expect information transfer only in case of a mismatch between top-down input from V1 and bottom-up input from the RGC. Hence, the LGN should spike whenever it received a spike exclusively in the top-down signal *or* in the bottom-up signal. This is measured by the synergistic information, *I*_*syn*_(*LGN*^+^: *RGC*^−^, *V*1^−^). If the synapse coded for predictable input, we would expect information transfer in case of matching inputs, i.e., the LGN should spike whenever it received a spike in *both* input signals. This is measured by the shared information, *I*_*shd*_(*LGN*^+^: *RGC*^−^, *V*1^−^).

### Considerations on cortico-cortical predictive coding and on scaling the current approach to neural populations

We remark that the results from the subcortical visual system presented here should not be seen as a refutation of predictive coding theories that propose the signaling of errors as a general information processing principle in the *neocortex* [21–23].

The analysis presented here relied on the fact that we were able to record the relevant inputs and outputs of a single neuron in a subcortical system. When transferring our analysis framework to cortico-cortical predictive coding, we face two difficulties: first, it will become next to impossible to cover *all* relevant inputs to a neuron with sufficient spatio-temporal resolution (although some in-vivo single-cell optical techniques hold some promise here); second, scaling the estimation of the resulting high-dimensional probability distributions will certainly pose an extreme challenge. At present, the best way forward here seems to rely on summary signals such as local field potentials (LFP) or optical techniques with a coarser resolution. We would then no longer be in a position to analyze the information transferred through a single neuron, and instead would have to resort to analyzing a triplet of cortical patches: one hierarchically lower patch that provides the inputs on which to quantify the *lAIS*, a second patch at an intermediate stage in the hierarchy that serves as a receiver, and the information transfer from this second patch to a third one even higher up in the processing hierarchy that provides a measure of information transferred in the outputs of the intermediate, second patch. This idea is presented in more detail in [5].

Despite these difficulties, the analysis of cortico-cortical predictive coding using the proposed information-theoretic framework seems highly promising, as very explicit predictions on the type of predictive coding, the location of error-computing units in upper cortical layers, and the corresponding LFP-frequency signatures of error signal have been made [94, 95]. Thus, we expect these hypotheses to be directly testable using frequency-resolved measures of information transfer [96, 97].

## Conclusion

Tests of predictive coding theories are at risk of being influenced by implicit assumptions of researchers about what a brain should predict. To circumvent this, careful experimental designs are necessary but may not always be possible due to a lack of the required prior knowledge about brain function. Also, such tests cannot refute or confirm predictive coding theories based on data from other experiments not designed with predictive coding in mind—although such tests need to be performed for a theory that claims a rather broad applicability to brain function. For these reasons, an analysis framework that is independent of experimental design and the experimenter’s assumptions would be highly beneficial. Here we present such a framework based on the correlation between the information-theoretic equivalents of predictability and prediction errors. In a proof-of-principle analysis of the re-encoding of retinal ganglion cell inputs in the lateral geniculate nucleus principal cells we demonstrate coding for predictable information in an anesthetized animal.

## Supporting information

S1 Text Localized bias Panzeri-Treves-correction for plug-in estimators. (PDF)

S2 Text Toy example to demonstrate negative local storage-transfer correlations (LSTC). (PDF)

S1 Table Spike train statistics. Contribution and efficacy values are taken from [8].
(PDF)

S2 Table Optimized embedding lengths and information transfer delays. Optimized non-uniform embedding lengths for *lAIS* and *lTE* estimation and reconstructed information-transfer delay *u* for *lTE* estimation.
(PDF)

S3 Table Local storage-transfer correlation coefficients. Local storage-transfer correlation (LSTC) coefficients for all cell pairs with significant *lAIS* and *lTE*.
(PDF)

S4 Table Classification accuracy of relayed versus non-relayed RGC spikes from *lAIS*. Classification accuracy (ACC) of a k-nearest neighbor classifier (*k* = 5) that predicts whether a spike is relayed from either its *lAIS* values, the number of RGC spikes in a time window of 30 ms prior to the spike, or the preceding inter-spike interval (ISI), respectively (average classification accuracy over ten repetitions of a five-fold cross validation, ± one standard deviation, SD). The first column shows the accuracy of a baseline classifier (BL) that randomly predicts a relayed spike with a probability corresponding to the relative frequency of relayed spikes in the training data. Classification accuracy was similar between the classifiers trained on the three inputs. All trained models outperformed the baseline model. For all cell pairs, the prediction accuracy based on past RGC spiking activity was higher or equal to the prediction accuracy based on the *lAIS*. Our results show that based on a spike’s *lAIS*, it can be predicted whether that spike gets relayed. Such a prediction is, however, equally possible based on the spiking statistics (spike rates and ISI), underlying the *lAIS* estimate.
(PDF)

S1 Fig Distribution of optimized past variable lags. Distribution of lags of past variables identified through optimization of the non-uniform embedding for (A) *lAIS*, (B) *lTE* (source), and (C) *lTE* target.
(PDF)
