# Supplementary material for: Information-theoretic analyses of neural data to minimize the effect of researchers’ assumptions in predictive coding studies
Source: PLoS Comput Biol. 2023 Nov 17;19(11):e1011567. doi: 10.1371/journal.pcbi.1011567 (PMC10703417; doi:10.1371/journal.pcbi.1011567)
Supplement: S1 Text — (PDF) [file pcbi.1011567.s001.pdf]

Localized bias Panzeri-Treves-correction for plug-in estimators

The bias of plug-in entropy-estimators for finite samples sizes can be analytically approximated for asymptotic sampling regimes, i.e., $N \geq |\mathcal{A}_X|$, as shown by Panzeri and Treves [1, 2]:

$$B_X = BIAS\left[H(X)\right] = \frac{-1}{2N\ln(2)}\left[m-1\right], \tag{1}$$

$$B_{X|Y} = BIAS\left[H(X|Y)\right] = \frac{-1}{2N\ln(2)}\left[\sum_{y\in\mathcal{A}_Y} m_y - 1\right], \tag{2}$$

where m is the alphabet size $m = |\mathcal{A}_X|$, and m_y is the alphabet size given $Y = y$ has occurred, $m_y = |\mathcal{A}_{X|Y=y}|$.

Note that the true alphabet sizes, m and m_y, are typically not known for experimental data and the number of actually observed responses can only be considered a lower bound on the true values. An estimate of m and m_y can be obtained from experimental data via a *Bayesian counting procedure* [2, 3], for example implemented in the pyEntropy toolbox [4].

To obtain a local bias correction for $lAIS$ and lTE using the Panzeri-Treves correction, we first provide a bias correction for local MI. We start by applying the correction to a plug-in estimator for the non-local MI (Eq 1, main text),

$$\hat{I}(X;Y) \approx \sum_{y\in\mathcal{A}_Y}\sum_{x\in\mathcal{A}_X}\left[\hat{p}(x,y)\log_2\frac{\hat{p}(x,y)}{\hat{p}(x)p(y)}\right] + B_X - B_{X|Y}. \tag{3}$$

To obtain a localized version of this estimator, note that the correction term B_X is constant over $x \in \mathcal{A}_X$ and $y \in \mathcal{A}_Y$, but $B_{X|Y}$ is not. The latter can be rewritten as a sum over $y \in \mathcal{A}_Y$, $B_{X|Y} = \sum_{y\in\mathcal{A}_Y} b_{X|y} = \sum_{y\in\mathcal{A}_Y}\frac{-1}{2N\ln(2)}\left[m_y - 1\right]$, (Eq. 2), such that we can write

$$I(X;Y) \approx \sum_{y\in\mathcal{A}_Y}\left[\sum_{x\in\mathcal{A}_X}\left[\hat{p}(x,y)\log_2\frac{\hat{p}(x,y)}{\hat{p}(x)p(y)}\right] - b_{X|y}\right] + B_X, \tag{4}$$

where $b_{X|y}$ is the individual contribution of realization $y \in \mathcal{A}_Y$ to the average correction term $B_{X|Y}$. By dividing by the alphabet size m, we can rewrite this as

$$I(X;Y) \approx \sum_{y\in\mathcal{A}_Y}\left[\sum_{x\in\mathcal{A}_X}\left[\hat{p}(x,y)\log_2\frac{\hat{p}(x,y)}{\hat{p}(x)p(y)} - \frac{b_{X|y}}{m}\right]\right] + B_X. \tag{5}$$

We may now rewrite the corrected MI as the expected value over all observations, analogous to [5]. The constant term B_X can be brought inside the average because of the linearity of the expected value,

$$\begin{aligned}
I(X;Y) &\approx \sum_{y\in\mathcal{A}_Y, x\in\mathcal{A}_X}\left[\hat{p}(x,y)\log_2\frac{\hat{p}(x,y)}{\hat{p}(x)p(y)} - \frac{b_{X|y}}{m}\right] + B_X \\
&= \sum_{y\in\mathcal{A}_Y, x\in\mathcal{A}_X}\left[\frac{1}{N}\sum_{g=1}^{c(x,y)}\log_2\frac{\hat{p}(x,y)}{\hat{p}(x)p(y)} - \frac{b_{X|y}}{m}\right] + B_X \\
&= \frac{1}{N}\sum_{i=1}^{N}\left[\log_2\frac{\hat{p}(x,y)}{\hat{p}(x)p(y)} - \frac{b_{X|y}}{m}\right] + B_X \\
&= \left\langle i(x_n;y_n) - \frac{b_{X|y_n}}{m} + B_X\right\rangle_n .
\end{aligned} \tag{6}$$

From this expression, we can immediately formulate the PT-corrected estimators for $lAIS$,

$$
\begin{aligned}
AIS(X,n,j) &= \left\langle lAIS^{PT}(X,n) \right\rangle_n \\
&= \left\langle i(\mathbf{x}_{n-1};x_n) + B_{\mathbf{X}_{n-1}} - \frac{b_{\mathbf{X}_{n-1}|x_n}}{|\mathcal{A}_{\mathbf{X}_{n-1}}|} \right\rangle_n ,
\end{aligned} \tag{7}
$$

and for lTE (again because of the linearity of the expected value),

$$
\begin{aligned}
TE_{SPO}(X \rightarrow Y,u) &= \left\langle lTE^{PT}_{SPO}(X \rightarrow Y,u) \right\rangle_n \\
&= I\left(Y_n; \mathbf{X}_{n-u}|\mathbf{Y}_{n-1}\right) \\
&= \hat{I}\left(\mathbf{X}_{n-u}; Y_n, \mathbf{Y}_{n-1}\right) + B_{\mathbf{X}_{n-u}} - B_{\mathbf{X}_{n-u}|Y_n,\mathbf{Y}_{n-1}} \\
&\quad - \hat{I}\left(\mathbf{X}_{n-u}; \mathbf{Y}_{n-1}\right) - B_{\mathbf{X}_{n-u}} + B_{\mathbf{X}_{n-u}|\mathbf{Y}_{n-1}} \\
&= \Bigg\langle i(\mathbf{x}_{n-u}; y_n, \mathbf{y}_{n-1}) + B_{\mathbf{X}_{n-u}} - \frac{b_{\mathbf{X}_{n-1}|y_n,\mathbf{y}_{n-1}}}{|\mathcal{A}_{\mathbf{X}_{n-u}}|} \\
&\quad - i(\mathbf{x}_{n-u}; \mathbf{y}_{n-1}) + B_{\mathbf{X}_{n-u}} - \frac{b_{\mathbf{X}_{n-1}|\mathbf{y}_{n-1}}}{|\mathcal{A}_{\mathbf{X}_{n-u}}|} \Bigg\rangle_n .
\end{aligned} \tag{8}
$$

In the present analysis, relative average bias correction for $lAIS$ ranged from $-3.11\,\%$ to $13.54\,\%$, and for lTE ranged from $0.03\,\%$ to $1.28\,\%$ (see Table 1 for mean corrections for all pairs).

Table 1: Mean relative local bias correction for $lAIS$ and lTE estimates (SD denotes the standard deviation).

Pair	$lAIS$		lTE	
	mean	SD	mean	SD
1	0.0261	0.0594	0.00589	0.0027
2	0.0221	0.0253	0.00336	0.0033
3	0.0266	0.0457	0.00215	0.0033
4	0.1053	0.0585	0.00081	0.0006
6	-0.0311	0.0562	0.00579	0.0042
7	0.0906	0.0334	0.00167	0.0040
8	0.0575	0.0628	0.00186	0.0008
9	0.0831	0.0369	0.01282	0.0155
10	0.0352	0.1881	0.00150	0.0119
11	0.0352	0.1881	0.00572	0.0242
12	0.0239	0.0070	0.00137	0.0005
13	0.0421	0.0262	0.00029	0.0004
14	0.1354	0.1241	0.00336	0.0197
15	0.0240	0.0065	0.00304	0.0012
16	0.0353	0.0172	0.00278	0.0011
17	0.0233	0.0047	0.00992	0.0019

Furthermore, we tested whether our estimation procedure including the applied bias correction suffered from fluctuations due to the number of samples used for analysis. We repeated the analysis on the first and second half on data from cell pairs 6 and 10. For pair 10, the LSTC deviated from the LSTC of the full recording, 0.2675 ($p = 0.0000$), by 0.0074 and -0.0132, respectively. For pair 6, the LSTC deviated from the LSTC of the full recording, 0.0149 ($p = 0.0000$), by -0.0070 and 0.0004.

References

[1] G M. Note on the bias of information estimates. In: Information Theory in Psychology II-B. Glencoe, IL: Free Press; 1955. p. 95–100.

[2] Panzeri S, Treves A. Analytical estimates of limited sampling biases in different information measures. Network: Computation in Neural Systems. 1996;7:87–107.

[3] Panzeri S, Senatore R, Montemurro MA, Petersen RS. Correcting for the sampling bias problem in spike train information measures. Journal of neurophysiology. 2007;98(3):1064–1072.

[4] Ince RAA, Petersen RS, Swan DC, Panzeri S. Python for Information Theoretic Analysis of Neural Data. Frontiers in Neuroinformatics. 2009;3(4).

[5] Lizier JT. The local information dynamics of distributed computation in complex systems. Berlin, Heidelberg: Springer Science & Business Media; 2013.
