# Supplementary material for: Information-theoretic analyses of neural data to minimize the effect of researchers’ assumptions in predictive coding studies
Source: PLoS Comput Biol. 2023 Nov 17;19(11):e1011567. doi: 10.1371/journal.pcbi.1011567 (PMC10703417; doi:10.1371/journal.pcbi.1011567)
Supplement: S2 Text — (PDF) [file pcbi.1011567.s002.pdf]

Toy example to demonstrate negative local storage-transfer correlations (LSTC)

To demonstrate that negative local storage-transfer correlations (LSTC) are possible, we generated a toy example by simulating artificial LGN responses based on RGC inputs. In particular, we defined a threshold, t_{ISI}, and for each RGC spike with a prior inter-spike interval (ISI) lower than the threshold, we ensured that there was no response in the LGN. Hence, we removed the effect found in the actual data, where the second spike in a tuple was highly predictable from its own past activity and more often triggered a response in the LGN. In our simulated data, the second spike in a tuple was still highly predictable, but we prevented the LGN from firing in response. Figure 1 shows results for pair 4, for four thresholds and different numbers of samples taken from the beginning of the actual recordings. For $t_{ISI} \geq 8$, we found small, negative LSTC with similar magnitude to correlations found in the original data. Correlations were statistically significant as established using a permutation test with 200 permutations.

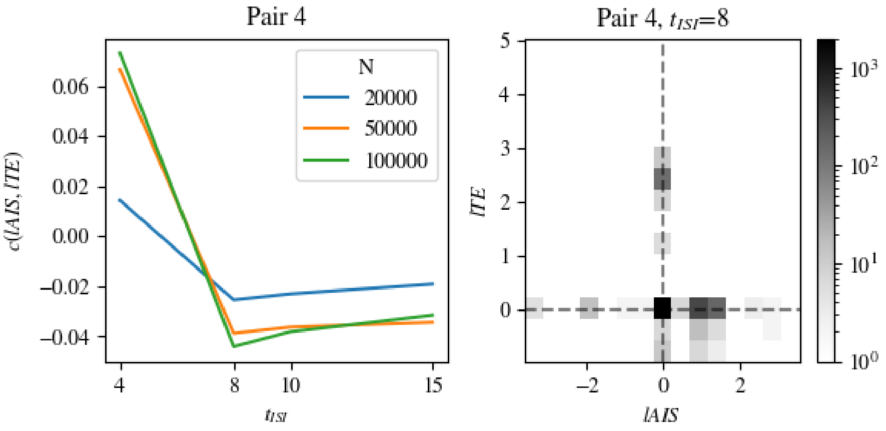

Figure 1: **Local storage-transfer correlation for simulated data based on recordings from pair 4.** Pearson correlation between local active information storage, $lAIS$, and local transfer entropy, lTE, for varying numbers of samples and thresholds, 4, 8, 10 and 15 (left). Histogram of joint $lAIS$ and lTE distribution for pair 4 ($N = 100000$), $t_{ISI} = 8$ ($c(lAIS, lTE) = -0.04413^{**}$).
