# Supplementary material for: Information-theoretic analyses of neural data to minimize the effect of researchers’ assumptions in predictive coding studies
Source: PLoS Comput Biol. 2023 Nov 17;19(11):e1011567. doi: 10.1371/journal.pcbi.1011567 (PMC10703417; doi:10.1371/journal.pcbi.1011567)
Supplement: S1 Table — Contribution and efficacy values are taken from [8]. (PDF) [file pcbi.1011567.s003.pdf]

Pair	N	delay[a]	RGC		LGN		efficacy[b]	contribution[c]
			no. spikes	rate	no. spikes	rate		
1	710,597	3	20,418	0.029	7,357	0.010	5.6	15.4
2	709,555	3	40,316	0.057	3,849	0.005	4.1	42.5
3	710,302	2	31,064	0.044	8,179	0.012	13.8	52.6
4	709,019	2	20,267	0.029	9,830	0.014	15.9	32.8
5	2,374,622	2	86,000	0.036	22,212	0.009	1.7	6.4
6	471,740	2	18,200	0.039	3,055	0.006	1.7	10.3
7	709,014	3	9,925	0.014	6,410	0.009	2.9	4.5
8	710,181	3	29,057	0.041	9,239	0.013	3.6	11.4
9	710,861	3	15,800	0.022	4,126	0.006	1.7	6.6
10	710,892	3	39,169	0.055	4,788	0.007	9.3	75.9
11	710,892	3	39,169	0.055	3,174	0.004	5.0	61.6
12	1,186,696	6	11,816	0.010	22,141	0.019	4.8	2.6
13	709,706	2	23,048	0.032	20,982	0.030	34.6	38.0
14	374,866	2	25,139	0.067	6,593	0.018	4.9	24.6
15	710,723	2	8,979	0.013	8,858	0.012	2.4	2.4
16	710,823	2	15,901	0.022	30,151	0.042	13.0	6.8
17	471,876	4	4,597	0.010	2,139	0.005	10.8	25.2

[a] Information-transfer delay between RGC and lGN, reconstructed during transfer entropy estimation. [b] Percentage of RGC spikes preceding an LGN spike. [c] Percentage of LGN spikes preceded by RGC spikes.
