# Supplementary material for: Information-theoretic analyses of neural data to minimize the effect of researchers’ assumptions in predictive coding studies
Source: PLoS Comput Biol. 2023 Nov 17;19(11):e1011567. doi: 10.1371/journal.pcbi.1011567 (PMC10703417; doi:10.1371/journal.pcbi.1011567)
Supplement: S2 Table — Optimized non-uniform embedding lengths for lAIS and lTE estimation and reconstructed information-transfer delay u for lTE estimation. (PDF) [file pcbi.1011567.s004.pdf]

Cell Pair	1	2	3	4	6	7	8	9	10	11	12	13	14	15	16	17
$lAIS$	8	9	9	9	7	8	9	7	10	10	6	8	7	4	7	4
lTE source	4	4	4	2	3	1	2	3	4	5	1	2	2	1	3	3
lTE target	5	6	7	9	3	7	7	4	7	5	8	7	6	6	5	5
delay u	3	3	2	2	2	3	3	3	3	3	6	2	2	2	2	4
