# Supplementary material for: Information-theoretic analyses of neural data to minimize the effect of researchers’ assumptions in predictive coding studies
Source: PLoS Comput Biol. 2023 Nov 17;19(11):e1011567. doi: 10.1371/journal.pcbi.1011567 (PMC10703417; doi:10.1371/journal.pcbi.1011567)
Supplement: S3 Table — Local storage-transfer correlation (LSTC) coefficients for all cell pairs with significant lAIS and lTE. (PDF) [file pcbi.1011567.s005.pdf]

Cell Pair	$c(LAIS, LTE)$	p-value	contribution [%]
1	0.0454	0.0000	15.40
2	0.0485	0.0000	42.50
3	0.1821	0.0000	52.60
4	0.1878	0.0000	32.80
6	0.0149	0.0000	10.30
7	0.0285	0.0000	4.50
8	0.0536	0.0000	11.40
9	0.0331	0.0000	6.60
10	0.2675	0.0000	75.90
11	0.1884	0.0000	61.60
12	0.0695	0.0000	2.60
13	0.0443	0.0000	38.00
14	-0.0209	1.0000	24.60
15	0.0056	0.0040	2.40
16	0.1866	0.0000	6.80
17	0.0827	0.0000	25.20

$c(LSTC, contribution) = 0.6879$, $p = 0.0030$
