# Supplementary material for: Information-theoretic analyses of neural data to minimize the effect of researchers’ assumptions in predictive coding studies
Source: PLoS Comput Biol. 2023 Nov 17;19(11):e1011567. doi: 10.1371/journal.pcbi.1011567 (PMC10703417; doi:10.1371/journal.pcbi.1011567)
Supplement: S4 Table — Classification accuracy (ACC) of a k-nearest neighbor classifier (k = 5) that predicts whether a spike is relayed from either its lAIS values, the number of RGC spikes in a time window of 30 ms prior to the spike, or the preceding inter-spike interval (ISI), respectively (average classification accuracy over ten repetitions of a five-fold cross validation, ± one standard deviation, SD). The first column shows the accuracy of a baseline classifier (BL) that randomly predicts a relayed spike with a probability corresponding to the relative frequency of relayed spikes in the training data. Classification accuracy was similar between the classifiers trained on the three inputs. All trained models outperformed the baseline model. For all cell pairs, the prediction accuracy based on past RGC spiking activity was higher or equal to the prediction accuracy based on the lAIS. Our results show that based on a spike’s lAIS, it can be predicted whether that spike gets relayed. Such a prediction is, however, equally possible based on the spiking statistics (spike rates and ISI), underlying the lAIS estimate. (PDF) [file pcbi.1011567.s006.pdf]

Pair	ACC BL	ACC $lAIS$ $\pm SD$	ACC RGC spikes $\pm SD$	ACC ISI $\pm SD$
1	91.9	95.5 $\pm$ 0.28	**95.5** $\pm$ 0.29	95.5 $\pm$ 0.29
2	93.9	96.9 $\pm$ 0.19	**96.9** $\pm$ 0.19	96.9 $\pm$ 0.19
3	79.0	88.1 $\pm$ 0.37	**88.1** $\pm$ 0.38	88.1 $\pm$ 0.38
4	78.8	87.9 $\pm$ 0.49	**87.9** $\pm$ 0.48	87.9 $\pm$ 0.47
6	96.5	**98.2** $\pm$ 0.19	**98.2** $\pm$ 0.19	98.2 $\pm$ 0.19
7	93.2	96.5 $\pm$ 0.31	**96.5** $\pm$ 0.30	96.1 $\pm$ 0.35
8	92.6	96.1 $\pm$ 0.19	**96.1** $\pm$ 0.19	96.1 $\pm$ 0.19
9	96.3	**98.1** $\pm$ 0.21	**98.1** $\pm$ 0.21	98.1 $\pm$ 0.21
10	84.3	91.4 $\pm$ 0.28	**91.4** $\pm$ 0.28	91.4 $\pm$ 0.28
11	91.2	95.4 $\pm$ 0.20	**95.4** $\pm$ 0.20	95.4 $\pm$ 0.20
12	90.4	94.9 $\pm$ 0.48	**95.0** $\pm$ 0.48	94.9 $\pm$ 0.50
13	60.4	72.8 $\pm$ 0.51	**72.9** $\pm$ 0.51	72.8 $\pm$ 0.52
14	92.2	95.9 $\pm$ 0.24	**95.9** $\pm$ 0.24	95.9 $\pm$ 0.24
15	93.8	**96.8** $\pm$ 0.36	**96.8** $\pm$ 0.36	96.8 $\pm$ 0.37
16	74.8	85.2 $\pm$ 0.62	**85.2** $\pm$ 0.61	85.2 $\pm$ 0.61
17	85.0	91.9 $\pm$ 0.85	**91.9** $\pm$ 0.85	91.8 $\pm$ 0.88
