# Supplementary figures and images for: Information-theoretic analyses of neural data to minimize the effect of researchers’ assumptions in predictive coding studies
Source: PLoS Comput Biol. 2023 Nov 17;19(11):e1011567. doi: 10.1371/journal.pcbi.1011567 (PMC10703417; doi:10.1371/journal.pcbi.1011567)

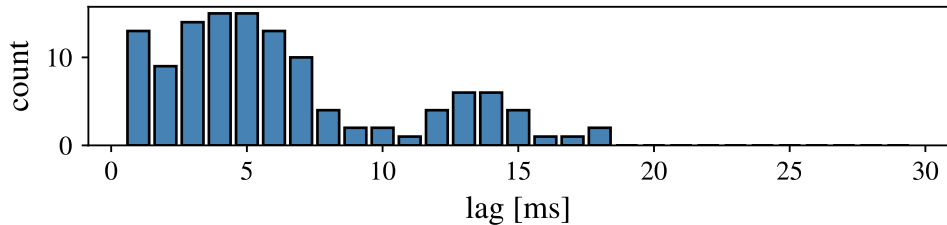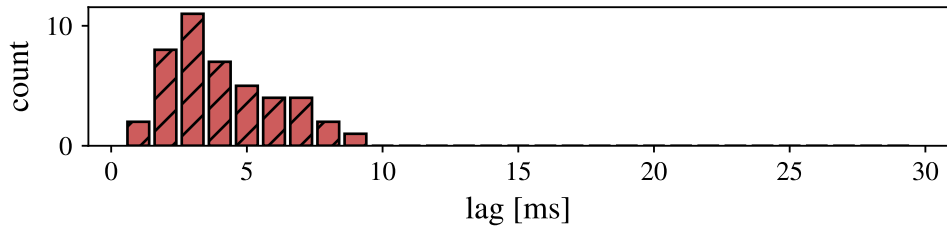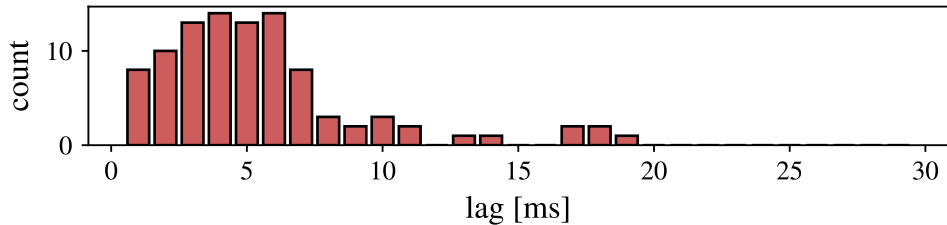

Supplement: S1 Fig — Distribution of lags of past variables identified through optimization of the non-uniform embedding for (A) lAIS, (B) lTE (source), and (C) lTE target. (PDF) [file pcbi.1011567.s007.pdf]
